# Differences in the temporal scale of reproductive investment across the slow‐fast continuum in a passerine

**DOI:** 10.1111/ele.13982

**Published:** 2022-03-02

**Authors:** Samantha C. Patrick, Denis Réale, Jonathan R. Potts, Alastair J. Wilson, Claire Doutrelant, Céline Teplitsky, Anne Charmantier

**Affiliations:** ^1^ 4591 School of Environmental Sciences University of Liverpool Liverpool UK; ^2^ Département des Sciences Biologiques Université du Québec A Montréal Québec Canada; ^3^ School of Mathematics and Statistics University of Sheffield Sheffield UK; ^4^ Centre for Ecology and Conservation University of Exeter (Penryn Campus) Cornwall UK; ^5^ CEFE Univ Montpellier CNRS EPHE IRD Montpellier France

**Keywords:** blue tits, carry‐over effects, life‐history, pace‐of‐life, senescence

## Abstract

Life‐history strategies differ with respect to investment in current versus ‘future’ reproduction, but when is this future? Under the novel ‘temporality in reproductive investment hypothesis’, we postulate variation should exist in the time frame over which reproductive costs are paid. Slow‐paced individuals should pay reproductive costs over short (e.g. inter‐annual) time scales to prevent reproductive costs accumulating, whereas fast‐paced individuals should allow costs to accumulate (i.e. senescence). Using Fourier transforms, we quantify adjustments in clutch size with age, across four populations of blue tits (*Cyanistes caeruleus*). Fast populations had more prevalent and stronger long‐term changes in reproductive investment, whereas slower populations had more prevalent short‐term adjustments. Inter‐annual environmental variation partly accounted for short‐, but not long‐term changes in reproductive investment. Our study reveals individuals differ in when they pay the cost of reproduction and that failure to partition this variation across different temporal scales and environments could underestimate reproductive trade‐offs.

## Graphical abstract

The temporality in reproductive investment hypothesis, proposed here for the first time, predicts that individuals will pay the costs of reproduction over different time scales. Our results show blue tits with a faster pace‐of‐life delay paying the costs of reproduction where as slow individuals pay the costs of short time scales and avoid them accumulating in later life.

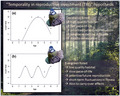

## INTRODUCTION

Life‐history (LH) theory predicts how organisms prioritise the allocation of resources (Stearns, 1989, 1992). Work in this field has often focused on longevity and lifetime survival and/or reproductive success with the prediction that when resources are limited, species, populations and individuals face a trade‐off between current and future reproduction, as well as between reproduction and survival. The covariation between LH traits can be used to place animals along a continuum from a fast to a slow pace‐of‐life (POL; Ricklefs & Wikelski, 2002; Stearns, 1992). Differences in the POL are linked to ecological conditions and intrinsic state (McNamara & Houston, 1996; Mueller et al., 1991; Ricklefs, 1991), likely driven by variation in resources (King et al., 2011; van Noordwijk & Jong, 1986). A fast LH will be associated with an increased allocation to current reproduction at a cost of survival and future reproduction, whereas a slow LH correlates with increased allocation to current survival, and a reproductive effort more evenly distributed over a longer lifetime. Such variation is captured between species in classic r/K selection theory (Pianka, 1970; Roff, 1992) but is also proposed to exist among individuals and populations. These principals may be directly applied at the individual level and by simplifying analyses to the single trade‐off between current and future reproduction, theoretical predictions are most robust (Araya‐Ajoy et al., 2018; Dammhahn et al., 2018; Del Giudice, 2020; Mathot & Frankenhuis, 2018).

As reproduction is costly and resources limited, allocation to current reproduction reduces the resources available for survival and hence future reproduction (Stearns, 1989). However, estimating survival itself can be challenging in the wild and many studies measure the change in reproductive investment over time, based on the assumption that there will be a negative covariation between allocation to current reproduction and survival (e.g. Bouwhuis et al., 2009; Hamel et al., 2014; Nussey et al., 2007; Reid et al., 2003). Theoretical and empirical research has shown that there is among‐individual heterogeneity in reproductive investment over predefined time scales (e.g. annual or long‐term such as lifetime). For example, over the long term, individuals that invest heavily in reproduction in early‐life experience a stronger cost to late‐life reproductive performance (i.e. a stronger senescent decline; Charlesworth, 2001; Kirkwood & Rose, 1991; Nussey et al., 2008; Williams et al., 2006). However, short‐term effects are also important and widely studied as carry‐over effects, where reproductive performance in one year incurs costs that impact on the next breeding attempt (Harrison et al., 2011). These short‐term changes in reproductive investment can be indicative of a reproductive cost but might also arise from temporal variation in environmental conditions, possibilities that can be hard to disentangle empirically. No study in the wild has assessed whether individuals, populations or species with different LH strategies differ in the temporal structure of reproductive costs, experienced over short and long periods. Here, we formulate the ‘temporality in reproductive investment **(**TRI**)**’ hypothesis (Table 1), which predicts that slow *versus* fast individuals will pay the cost of reproduction over different time scales.

**TABLE 1:** Definitions of terms used throughout the manuscript

	Definition
Temporality in reproductive investment (TRI) hypothesis	The hypothesis predicting that individuals will differ in when they pay the costs of reproduction and, hence, the temporal scale at which changes in reproductive investment are detected
Reproductive investment curve (RIC)	A Fourier mode estimated from a fast‐Fourier transform, in this case study, applied to clutch size. This curve represents a change in reproductive investment over time.
Strength/amplitude of RIC	The magnitude of a RIC measured as the difference between the peak and trough of the curve (twice the ‘mathematical’ amplitude)
Temporal scale of RIC	The wavelength of a RIC
Reproductive investment strategy	The observed tempo and strength of reproductive investment of an individual across its lifetime
Dominant RIC	The RIC with the greatest strength (amplitude)
Secondary RIC	The RIC with the second greatest strength (amplitude)
Short‐term RIC	An RIC with a temporal scale of 2 years
Long‐term RIC	A RIC with a temporal scale >2 years
Absolute clutch size	The actual clutch size measured in the wild per individual per year
Mean‐centred clutch size	The clutch size of an individual minus the population‐year average. This measure controls for inter‐annual environmental variation in clutch size in the local population, for example, due to density‐dependent or environment‐dependent effects on clutch size.
Mean individual clutch size	The mean clutch size of an individual across its lifetime, used as an indicator of individual quality

The fast‐slow LH framework can be extended to incorporate a variety of physiological and behavioural traits through the POL syndrome framework (Réale et al., 2010; Ricklefs & Wikelski, 2002). These suites of correlated traits predict that risk‐taking behaviour will increase age‐independent mortality risk, reducing the chances of an individual surviving until ‘later life’. Therefore, these faster individuals are likely to die before experiencing reproductive and actuarial senescence, and age‐specific mortality (i.e. mortality risk that increases with age, in line with senescence theory). If fast individuals survive the extrinsic mortality processes long enough to reach late life, the deferred costs should manifest as a steep senescent decline. Conversely, assuming individuals with a slow LH are risk avoiders, and hence have a higher age‐independent survival probability, they are predicted to allocate resources preferentially to future reproduction, as they have a greater chance of breeding in the future. An individual with a slow LH will show variable reproductive effort (and success) over successive breeding events as reproductive costs are paid in the short‐term. However, a slow LH should also be coupled with a shallower senescent decline than that predicted in fast individuals.

To date, in the context of POL syndromes, changes in reproductive investment have been largely modelled as long‐term declines, illustrated by the negative correlation between early and late life reproductive performance (Baudisch et al., 2013; Patrick & Weimerskirch, 2015; Réale et al., 2010). In contrast, existing studies have omitted short‐term changes, despite the integration of behavioural and LH traits within the POL syndrome framework providing robust predictions for TRI. The TRI hypothesis offers a mechanistic link explaining variation in LH strategy, which is intuitive and in line with the theories on how LH shapes senescence patterns (Kirkwood & Rose, 1991; Nussey et al., 2008), and individual differences in the POL (Réale et al., 2010). Differences in reproductive investment must be modelled simultaneously across different temporal scales to assess their relative importance among individuals.

As highlighted above, previous studies did not account for the possibility of among‐individual differences in the temporal scale over which reproductive costs are paid. Although there are suitable techniques to model reproductive investment over time using general linear models (Hamel et al., 2014; Nussey et al., 2008), these approaches are limited to (a) testing few temporally predefined time frames over which costs are paid and (b) by the availability of sufficient data. A more general and flexible approach to TRI would allow the temporal scales over which reproductive costs are paid within an individual’s lifetime to be directly estimated from empirical data. Modelling such effects simultaneously is imperative to decouple reproductive versus environmental changes.

**FIGURE 1:**
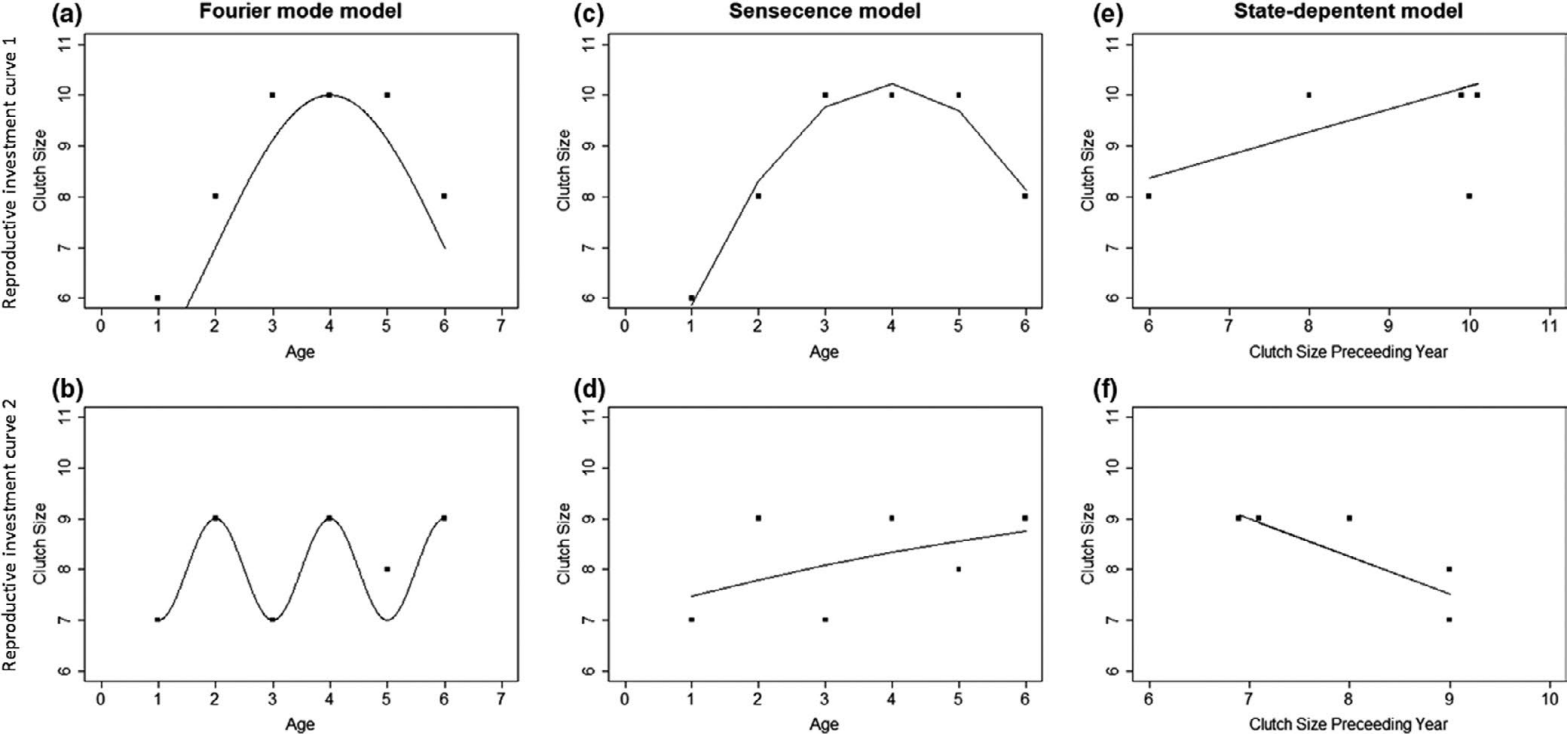
A schematic illustrating two simulated reproductive investment curves (RICs) occurring over different temporal scales. An RIC can vary in the temporal scale of changes in investment and in the magnitude of these changes (amplitude). RIC 1 (Panel a, c, e): An example where clutch size increases gradually over time until mid‐life when it begins to decline and clutch size ranges from 6–10. RIC 2 (Panel b, d, f): An example where clutch size oscillates inter‐annually and ranges from 7 to 9. RIC 1 shows a long‐term RIC with a high amplitude, whereas RIC 2 shows a short‐term RIC with a lower amplitude. Here, we compare two widely used methods for analysing reproductive investment with a novel Fourier mode analysis. (a) A Fourier mode of wavelength 14 explains 99% of the variation in clutch size (note only part of this wave is shown in the figure), whereas (c) a quadratic (senescence) model explains 97% and (e) a state‐dependent model with a one‐year lag explains 17% of the variation. (b) A Fourier mode with a wavelength of two explains 83% of the variation, whereas (d) a quadratic model explains 17% and (f) a state‐dependent model explains 70%. This figure shows a simplified example, which is likely not realistic in the wild. First, individuals may not have the same lifespan, and hence time series length, and this may covary with the temporal scale of RICs. Second, multiple RICs over different temporal scales (i.e. multiple Fourier modes) can, and are likely to, occur within an individual

Fourier analysis is based on the transformation of time series data into stationary sine waves (here termed reproductive investment curves (RICs); See Table 1 for definitions of terminology used in the manuscript) of varying wavelengths. Such methods have been used across ecology, to identify frequency patterns that explain ecological variation (Cazelles et al., 2008; Couteron et al., 2005; Polansky et al., 2010). In the current context, the peaks and troughs of the wave represent high and low reproductive values, and the difference between these – the amplitude – measures the change in reproductive investment across the defined time frame. The wavelength (time between successive peaks) is the temporal scale of changes in reproductive investment. This can be estimated for a partial wave (e.g. Figure 1a) or a full wave (e.g. Figure 1b). This mathematical approach can capture long‐term changes in a trait, previously modelled using, for instance, quadratic mixed models (Figure 1a,c) and short‐term changes, previously modelled, for example, using state‐dependent models (Figure 1b,f). Its strength lies in the ability to capture both short‐ and long‐term changes in a single analysis for an individual, without predefined temporal scales, something previous techniques have been unable to fully achieve.

Here, we quantify how reproductive investment changes over time within individual lifetimes and compare the temporal scale of these changes across four populations of blue tits, known to differ in their average LH (Charmantier et al., 2016; Dubuc‐Messier et al., 2017). We examine changes in clutch size (as a proxy of reproductive investment) with age for 847 individuals, breeding in either evergreen holm oak (*Quercus Ilex*; low productivity forest), or deciduous downy oak (*Quercus pubescens*; high productivity forest) habitats. We use Fourier transforms to estimate reproductive investment changes over time, based on the dominant wavelength (temporal scale) and amplitude (change in clutch size with associated scale, indicative of the accumulation of costs) for each individual, and compare these across populations. We examine both the absolute clutch size of individuals, indicative of the variation observed, and a population level mean‐centred measure of clutch size, which controls for inter‐annual environmental variation in clutch size in the local population.

We predict that, given birds in evergreen forests have a slower LH on average than those in deciduous forests (Bastianelli et al., 2021; Dubuc‐Messier et al., 2017), they will pay the cost of reproduction over shorter average time frames (short wavelengths). Birds in deciduous forests, which have a faster LH, will pay the cost of reproduction over longer time frames (long wavelengths), akin to senescence, as they will allow costs to accumulate across years, maintaining high reproductive performance in early years at the cost of steeper senescence subsequently. We predict that the higher the number of offspring now, the greater the cost to be paid in the future (irrespective of when). Therefore, fast individuals are predicted to have oscillations in reproductive success of greater amplitude and wavelength, the former arising from high reproductive effort with costs allowed to accumulate across reproductive events.

## MATERIALS AND METHODS

### Study site and systems

The blue tit (*Cyanistes caeruleus*) is an annually breeding small passerine found across Europe and the western Palaearctic (Stenning, 2018) with variable clutch size across the range. This study used four blue tit populations monitored in nest‐box study areas in France: (i) D‐Rouviere (Latitude 43.66N; Longitude 3.67W; near Montpellier) in a heterogeneous forest dominated by deciduous downy oaks, *Quercus pubescens*; (ii) D‐Muro (42.55N; 8.92W; in Corsica) dominated by deciduous downy oaks; (iii) E‐Muro (42.59N; 8.96W; in Corsica) dominated by evergreen holm oaks, *Quercus ilex*, and located 5.6 km from D‐Muro and iv) E‐Pirio (42.38N; 8.75W; in Corsica) composed of evergreen holm oaks and located 24.1 km from D‐Muro (Charmantier et al., 2016). Populations differ in their LH. Deciduous populations have a faster LH, including larger clutch size, higher fledging success (number of chicks fledging the nest), but lower juvenile recruitment (probability of a chick recruiting to the breeding population), and lower annual survival for breeding birds. Annual survival probability is typically 0.42–0.47 in deciduous populations, with a generation time of 1.91–2.00 years, compared with an annual survival probability of 0.55–0.57 in evergreen populations and a generation time of 2.64 years (estimated for E‐Pirio only; Bastianelli et al., 2021; Charmantier et al., 2004, 2016).

Population monitoring of nest‐boxes started between 1976 (E‐Pirio) and 1998 (E‐Muro). Breeding data until 2018 were used in this study including only birds that had the potential to breed for at least 3 years. We calculated age from birth year for chicks born in the population. For immigrants, we distinguished one‐year‐old birds from those aged two or more at first capture by plumage characteristics. The final data set contained birds aged 1–9 years (Charmantier et al., 2016; Supplementary material Appendix 1). Birds were sexed based on the presence of a brood patch or plumage characteristics (Stenning, 2018). We excluded all second clutches (2% of all breeding attempts) and replacement clutches (sometimes laid when birds fail early in the season – 10% of all breeding attempts) as they are likely to exhibit different RICs to first clutches. A bird that lays ten eggs in a single clutch will have a different reproductive investment to one that lays five eggs, fails, and then lays a replacement clutch of five eggs. While such differences are interesting and biologically important it is not possible to model them in the current analyses. Any possible polygamous pairs (<1% of breeding attempts) were also excluded. We restricted analyses to birds where we had a continuous time series removing any individual with missing breeding data in years they were alive (12%). We elected to do this as it is difficult to infer biological meaning from the years without (recorded) breeding – individuals may have skipped a year (i.e. reproductive investment of zero), which would be valuable data, but it is possible (and in our view probable) that more often they were breeding outside the nest‐box study area. The final sample sizes were as follows: D‐Rouviere – N = 292; D‐Muro – N = 144; E‐Muro – N = 107; E‐Pirio – N = 304.

### Statistical analysis

We used clutch size as a measure of reproductive effort (Pettifor et al., 2001; Tinbergen, 2004), and its pseudo‐Gaussian distribution allowed simple Fourier analysis to be conducted (See Table 1 for definitions of terms used). We used two measures of clutch size: (i) absolute clutch size – reproductive investment in egg laying, making no assumption on the availability of resources and (ii) the deviation from the mean population‐year clutch size (hereafter mean‐centred clutch size). This measure accounts for among‐population differences driven by inter‐annual variation in the environment. This measure allows us to test the hypothesis that short‐term RICs may be capturing environmental differences over short‐time frames but that long‐term RICs will be independent of this variation.

We decomposed the time series of each measure of clutch size for each individual using the spectral package (Seilmayer, 2019) in R (R. Development Core Team, 2018), which performs a fast Fourier transform of time series data. This is a standard method for extracting information about oscillatory time series by approximating them by an infinite series of sine wave segments (called Fourier modes) with differing wavelengths and amplitudes – see for example, Brigham (1988). In this case, the wavelength is the time, in years, between successive peaks and here we define the amplitude as the difference between the peak and trough of each RIC. While mathematically the amplitude of a wave is the deviation between the peak and the median value, here we use amplitude in the broader sense to refer to the magnitude of the change in clutch size (see Table 1). The wavelengths and amplitudes of each Fourier mode were extracted for each individual. We occasionally found three or more Fourier modes but only when birds had a relatively long time series (five years or more), so we focus on just the two dominant Fourier modes here (see Supplementary material Appendix 2). The dominant Fourier mode denotes the wavelength of the sine wave with the largest amplitude, and we call this the ‘dominant RIC’; likewise, we define the “secondary RIC” as the wavelength of the sine wave with the second‐largest amplitude. Fourier transforms cannot be used when an individual has only one or two reproductive attempts because there is no oscillatory signal in a time series of <3 data points. So only time series where birds bred three or more times were used in analyses (See Supplementary material Appendix 4 for details of birds breeding less than three times). This approach is robust to detecting reproductive investment strategies with short time series (see Supplementary material Appendix 2).

Temporality in reproductive investment strategies was assessed by grouping RICs into short‐term, a dominant wavelength of 2 years, and long‐term RICs, a wavelength of more than two. Thus, a short‐term pattern arises when high levels of investment in reproduction translate into reduced levels in the next year. We based this grouping on the distribution of wavelengths – there was a clear large peak at 2 years (see Supplementary material Appendix 5; results were consistent when using a cut‐off of <4 years; see Supplementary material Appendix 6). This classification was carried out for both the dominant and secondary RICs. Although individuals differ in which RIC is dominant, almost all individuals show both short‐term and long‐term RICs.

To understand whether the proportion of birds exhibiting dominant long‐term RICs varied across populations, we fitted a generalised linear model with (estimated) dominant individual reproductive investment strategy as the response. This was treated as a binary metric for short‐ term (2 years; 0) or long‐ term (greater than 2 years; 1) reproductive investment strategy at the individual level. In this study, most birds showed a short‐ and long‐term reproductive investment strategies, so we did not model the secondary strategy. Population, sex and mean individual clutch size, calculated across the full time series per individual, were fitted as fixed effects and birth year as a random intercept. We fitted mean individual clutch size to control for individual differences in the intercept driven by differences in individual ‘quality’. We ran two models – using the RICs extracted from absolute and mean‐centred clutch size:

Time scale of RIC_i_ = β_0_ + β_1_Population_i_ + β_2_Sex_i_ + β_3_Mean individual clutch size_i_ + Birth Year_i_ + e_i_.

where time scale of RIC_i_ is a binary measure of the scale of an individual bird *i’s* RIC where short‐term (2 years) = 0 and long‐term (>2 years) = 1. Birth year is fitted as a random effect.

These models examine the change in reproductive performance for known breeding attempts – that is, they do not model failure to breed or any metric of survival, similarly to widely used senescence models. Studies have shown the importance of modelling selective appearance and disappearance in senescence models (Bouwhuis et al., 2009; Hämäläinen et al., 2014; Nussey et al., 2011), but these did not appear to affect our results (See Supplementary material Appendix 2 for full discussion).

We then compared the amplitude of the oscillation in individuals between populations using a log‐transformed amplitude (absolute clutch size) or square root transformed amplitude (mean‐centred clutch size) in a general linear mixed model with a Gaussian error structure. We transformed data to ensure residuals followed a Gaussian distribution. We extracted the amplitude of the short‐ and long‐term RICs and ran separate models for each, fitting population, sex and mean individual clutch size per bird as independent variables and birth year as a random intercept. We ran four models: Using absolute clutch size data, we extracted the amplitude of an individual’s short‐term and long‐term RIC and then repeated this using mean‐centred clutch size:

Amplitude of RIC_i_ = β_0_ + β_1_Population_i_ + β_2_Sex_i_ + β_3_Mean individual clutch size_i_ + Birth Year_i_ + e_i_.

where amplitude of RIC_i_ is the amplitude of an individual bird *i’s* (i) short‐term RIC using absolute clutch size, (ii) a bird’s short‐term RIC using mean‐centred clutch size, (iii) a bird’s long‐term RIC using absolute clutch size and (iv) a bird’s long‐term RIC using absolute clutch size. Birth year is fitted as a random effect.

For all model selection, we used Akaike's Information Criterion (AIC), using the MuMIn package in R (Barton, 2009). For all models, examining both RIC length and amplitude, we fitted population, mean individual clutch size and sex. From these we selected models with a structure that minimised AIC. We kept models which were within two AIC units of the best‐fitting model and used weighted‐model averages to calculate parameter estimates (Burnham & Anderson, 2004). We compare parameter estimates and discuss the strength of results in the discussion. Random effects were estimated from full models. For plotting purposes, estimated marginal means with associated standard errors were extracted using the emmeans package in R (Lenth, 2018) based on the best‐fitting model, and, displayed on the original scale.

## RESULTS

### Number of reproductive investment curves

When examining the RIC based on absolute clutch size, 89% of the analysed individuals had at least two Fourier modes, and 100% using mean‐centred clutch size suggesting multiple RICs. Comparing the dominant and secondary RIC for all birds surviving to at least three years old showed that 91% had one short‐ and one long‐term RIC when considering absolute clutch size and 92% using mean‐centred. The remaining birds showed two long‐term RICs of differing temporal scale.

### Temporality in reproductive investment strategies

Overall, the dominant RIC of 44% of all individuals was short‐term (wavelength two years; 43% when considering RICs based on mean‐centred clutch size), whereby a high clutch size would be followed by a lower one the next year. For the 56% of all birds that had a long‐term dominant RIC (57% when considering mean‐centred clutch size), 13% exhibited an RIC of four years (18% using mean‐centred clutch size) and 32% of six years (28% using mean‐centred clutch size). Wavelengths were not constrained to be whole integers (Supplementary material Appendix 3), and no other wavelength occurred in >5% of individuals.

**FIGURE 2:**
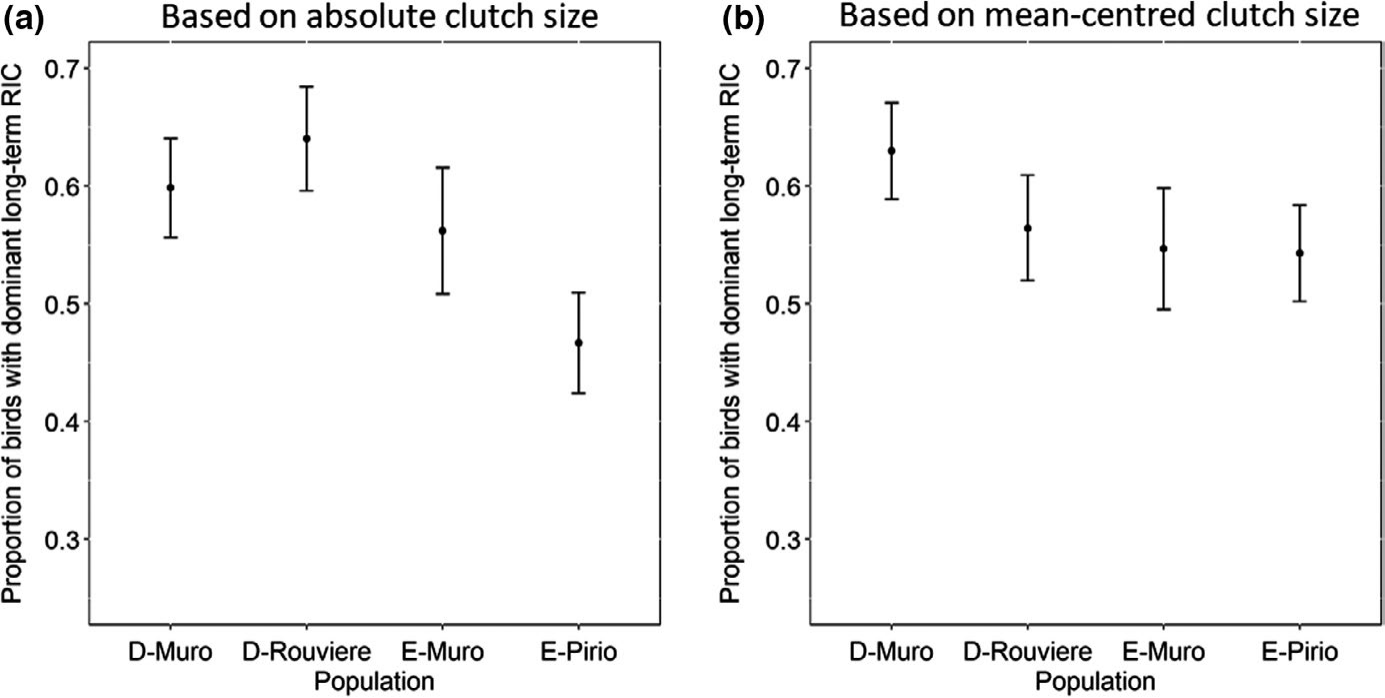
Reproductive investment strategies (RICs) among four blue tit populations, living in different habitats (D = deciduous oak; E = evergreen oak). Proportion of birds with a dominant (i.e. the wavelength with the highest amplitude) long‐term reproductive investment curve (wavelength of >2 years). (a) Using RICs calculated on absolute clutch size, E‐Pirio had the smallest proportion of birds with long‐term RICs and D‐Rouviere the largest. (b) Using RICs calculated on mean‐centred clutch size, E‐Pirio remained the population with the highest proportion of short‐term RICs, but D‐Muro was observed to have the lowest proportion of individuals with short‐term RICs, largely because the proportion of long‐term RICs in D‐Rouviere decreased. Plots shows estimated marginal means from a model including all fixed effects maintained in the best fitting models and associated ±1 standard errors on the original scale

Population was retained in the preferred models of the proportion of birds with long‐ *versus* short‐term RICs, providing some support for our prediction that the timescale of cost payment would differ among populations. E‐Pirio had the fewest individuals with dominant long‐term RICs and D‐Rouviere the most using absolute clutch size and estimates for these two populations had non‐overlapping standard errors. Using mean‐centred clutch size, E‐Pirio and E‐Muro had the fewest individuals with long‐term RICs and D‐Muro the most, but population differences were small (Table 2, Figure 2). Absolute clutch size models showed a weak negative effect of mean individual clutch size on the proportion of long‐term RICs, but when using mean‐centred clutch size this relationship was weakly positive. Males had a lower proportion of long‐term RICs, and this relationship was strongest using mean‐centred clutch size, but in all cases the standard errors overlapped considerably (Table 2, Figure 2).

**TABLE 2:** Results from the generalised linear mixed model examining the drivers of whether breeding blue tits show dominant short‐ or long‐term reproductive investment curves (RICs). Binary models where 0 = dominant RIC is short‐term (wavelength =2) and 1 = dominant RIC is long term (wavelength of >2). Absolute clutch size N = 787; mean‐centred clutch size N = 846. Birth year was included as a random effect in all models (Absolute clutch size: σ^2^ = 0.003; Mean‐centred clutch size σ^2 ^= 0.000). (a) Full model results and in bold the model with the minimum AIC, and all models with a delta AIC<2 which were selected. (b) Best‐fitting model(s) are shown with (averaged) parameter estimates and models weightings. Variables included in each model are marked with + and empty cells where variables were dropped. Results are shown using absolute clutch size and mean‐centred clutch size.

Intercept	Mean individual clutch size	Population	Sex	df	logLik	AICc	Delta	Weight
**(a)**								
Absolute clutch size								
**0.25**				**2**	**−539.26**	**1082.50**	**0.00**	**0.23**
**0.36**		+		**5**	**−536.49**	**1083.10**	**0.52**	**0.18**
**0.40**	**−0.17**	+		**6**	**−535.67**	**1083.40**	**0.90**	**0.15**
**0.25**	**0.07**			**3**	**−538.75**	**1083.50**	**0.98**	**0.14**
**0.28**			+	**3**	**−539.15**	**1084.30**	**1.78**	**0.10**
0.40		+	+	6	−536.35	1084.80	2.26	0.08
0.44	−0.17	+	+	7	−535.54	1085.20	2.68	0.06
0.29	0.07		+	4	−538.62	1085.30	2.76	0.06
Mean‐centred clutch size								
**0.39**			+	**3**	**−576.50**	**1159.00**	**0.00**	**0.29**
**0.39**	**0.09**		+	**4**	**−575.75**	**1159.50**	**0.51**	**0.22**
**0.28**				**2**	**−578.15**	**1160.30**	**1.28**	**0.15**
**0.67**		+	+	**6**	**−574.38**	**1160.90**	**1.84**	**0.11**
**0.28**	**0.08**			**3**	**−577.45**	**1160.90**	**1.90**	**0.11**
0.54		+		5	−576.19	1162.40	3.43	0.05
0.67	0.04	+	+	7	−574.33	1162.80	3.76	0.04
0.53	0.03	+		6	−576.15	1164.40	5.38	0.02

Parameter	Parameter estimate	Standard error	Parameter estimate	Standard error
	Absolute clutch size		Mean‐centred clutch size	
(b)				
Intercept^a^	0.31	0.14	0.39	0.16
D‐Rouviere	0.04	0.16	−0.03	0.11
E‐Muro	−0.03	0.19	−0.05	0.16
E‐Pirio	−0.17	0.27	−0.05	0.16
Mean individual clutch size	−0.02	0.11	0.03	0.06
Sex – male	−0.01	0.06	−0.18	0.17

^a^ D‐Muro is the population of reference and female the sex reference

### Amplitude of reproductive investment curves over different temporal scales

**FIGURE 3:**
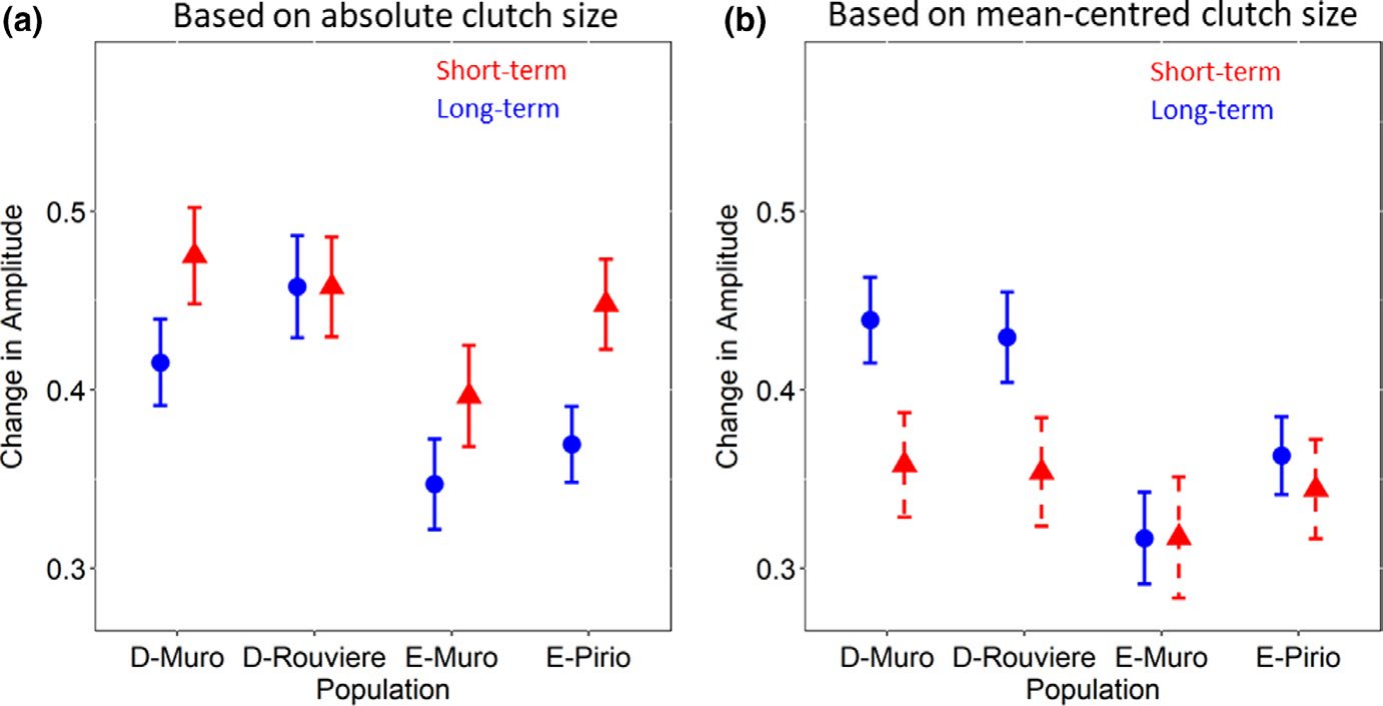
The wavelength amplitude associated with reproductive investment curves (RICs) among four blue tit populations, living in different habitats (D = deciduous oak; E = evergreen oak). (a) Results using absolute clutch size (b) Results using mean‐centred clutch size. Red =short‐term RICs; blue =long‐term RICs. Using absolute clutch size (a), deciduous populations had short‐term RICs with greater amplitude, but these differences between populations did not persist using mean‐centred clutch size (b) suggesting inter‐annual variation in environmental conditions is an important component of short‐term RICs. The amplitude of long‐term RICs was higher for birds in deciduous compared to evergreen woodlands using absolute clutch size (a), and these results were very similar when using mean‐centred clutch size (b), suggesting little effect of inter‐annual variation in environmental conditions among populations. Plots shows estimated marginal means from a model including all fixed effects maintained in the best‐fitting models and associated ±1 standard errors on the original scale. When population was maintained in the best‐fitting model solid lines are shown, and when it was dropped these effects are shown with dashed lines

When considering the amplitude of short‐term RICs, birds from faster, deciduous populations (D‐Muro and D‐Rouviere) had greater amplitude than E‐Muro and E‐Pirio using absolute clutch size, although these differences were relatively small. In contrast, there were no differences between the populations using mean‐centred clutch size, evident as population was no longer maintained in the best‐fitting model. This suggests an inter‐annual environmental component to short‐term RICs and an apparent decrease in amplitude (Table 3, Figure 3). Results comparing long‐term RICs showed strong evidence that birds from faster populations also showed greater amplitude in long‐term RICs using absolute clutch size and while the amplitude decreased slightly, these results persisted using mean‐centred clutch size, suggesting little effect of environmental variability (Table 4, Figure 3).

**TABLE 3:** Results from the general linear model examining variation in the amplitude of short‐term reproductive investment curves (wavelength =2 years), based on either absolute clutch size or mean‐centred clutch size (Absolute clutch size N = 633; mean‐centred clutch size N= 779). Models of amplitudes based on absolute clutch size were log‐transformed, and those based on mean‐centred clutch size square‐root transformed. (Absolute clutch size: σ^2^ = 0.001; mean‐centred clutch size σ^2 ^= 0.034) (a) Full model results and in bold the model with the minimum AIC, and all models with a delta AIC<2 which were selected. (b) Best‐fitting model(s) are shown with (averaged) parameter estimate and model weightings. Variables included in each model are marked with + and empty cells where variables were dropped. Results are shown using absolute clutch size and mean‐centred clutch size.

Intercept	Mean individual clutch size	Population	Sex	df	logLik	AICc	Delta	Weight
**(a)**								
Absolute clutch size								
**−0.88**	**0.06**		+	**5**	**−564.37**	**1138.80**	**0.00**	**0.46**
**−0.81**		+	+	**7**	**−562.73**	**1139.60**	**0.80**	**0.31**
−0.82	0.04	+	+	8	−562.34	1140.90	2.07	0.16
−0.88			+	4	−567.47	1143.00	4.17	0.06
−0.81	0.06			4	−569.73	1147.50	8.69	0.01
−0.74		+		6	−568.28	1148.70	9.87	0.00
−0.75	0.04	+		7	−567.85	1149.90	11.05	0.00
−0.81				3	−572.80	1151.60	12.81	0.00
Mean‐centred clutch size								
**0.56**			+	**4**	**−47.32**	**102.70**	**0.00**	**0.61**
**0.56**	**0.01**		+	**5**	**−47.03**	**104.10**	**1.44**	**0.30**
0.57		+	+	7	−46.54	107.20	4.54	0.06
0.57	0.00	+	+	8	−46.53	109.30	6.57	0.02
0.58				3	−53.08	112.20	9.50	0.01
0.59	0.01			4	−52.75	113.60	10.86	0.00
0.60		+		6	−52.35	116.80	14.11	0.00
0.60	0.00	+		7	−52.32	118.80	16.10	0.00

Parameter	Parameter estimate	Standard error	Parameter estimate	Standard error
	Absolute clutch size		Mean‐centred clutch size	
(b)				
Intercept^a^	−0.85	0.06	0.56	0.01
D‐Rouviere	0.00	0.04		
E‐Muro	−0.09	0.12		
E‐Pirio	−0.04	0.07		
Mean individual clutch size	0.04	0.03	0.00	0.01
Sex – male	0.16	0.05	0.06	0.02

^a^ D‐Muro is the population of reference and females the sex reference.

**TABLE 4:** Results from the general linear model examining variation in the amplitude of long‐term reproductive investment curves (wavelength >2 years), based on either absolute clutch size or mean‐centred clutch size (Absolute clutch size N = 633; mean‐centred clutch size N = 779). Models of amplitudes based on absolute clutch size were log‐transformed, and those based on mean‐centred clutch size square‐root transformed. Birth year was fitted as a random intercept in all models (absolute clutch size: σ^2^ = 0.007; mean‐centred clutch size σ^2^ = 0.000). (a) Full model results and in bold the model with the minimum AIC, and all models with a delta AIC<2, which were selected. (b) Best‐fitting model(s) are shown with (averaged) parameter estimates and model weightings. Variables included in each model are marked with + and empty cells where variables were dropped. Results are shown using absolute clutch size and mean‐centred clutch size.

Intercept	Mean individual clutch size	Population	Sex	df	logLik	AICc	Delta	Weight
**(a)**								
Absolute clutch size								
**−0.93**		+	+	**7**	**−563.69**	**1141.50**	**0.00**	**0.54**
**−0.94**	**0.02**	+	+	**8**	**−563.62**	**1143.50**	**1.92**	**0.21**
−0.97	0.10		+	5	−567.01	1144.10	2.56	0.15
−0.88		+		6	−566.86	1145.80	4.29	0.06
−0.88	0.02	+		7	−566.77	1147.70	6.17	0.02
−0.91	0.10			4	−569.87	1147.80	6.26	0.02
−0.97			+	4	−576.16	1160.40	18.83	0.00
−0.91				3	−578.94	1163.90	22.38	0.00
Mean‐centred clutch size								
**0.63**		+	+	**7**	**114.53**	**−214.90**	**0.00**	**0.72**
0.63	0.00	+	+	8	114.53	−212.90	2.04	0.26
0.60	0.03		+	5	108.78	−207.50	7.43	0.02
0.66		+		6	106.90	−201.70	13.22	0.00
0.66	0.00	+		7	106.93	−199.70	15.20	0.00
0.60			+	4	103.31	−198.60	16.35	0.00
0.62	0.03			4	101.60	−195.10	19.77	0.00
0.62				3	95.93	−185.80	29.08	0.00

Parameter	Parameter estimate		Standard error		Parameter estimate	Standard error
		Absolute clutch size			Mean‐centred clutch size	
(b)						
Intercept^a^		−0.94		0.06	0.63	0.02
D‐Rouviere		0.11		0.07	−0.01	0.02
E‐Muro		−0.19		0.09	−0.10	0.03
E‐Pirio		−0.13		0.07	−0.06	0.02
Mean individual clutch size		0.00		0.03		
Sex – male		0.12		0.05	0.06	0.02

^a^ D‐Muro is the population of reference and females the sex reference.

The amplitude of long‐term RICs did not appear to change when controlling for the environment (using mean‐centred clutch size), in keeping with the prediction that, as they are an accumulation of costs, they should be associated with a higher amplitude (Figure 3). Amplitude was greater for males for both short‐ and long‐time scales, and in both cases the sex difference was very weak when controlling for the environment. Mean individual clutch size was maintained in models of both the amplitude of short‐ and long‐term RICs, using both absolute and mean‐centred clutch size, but these relationships were very weak (Tables 3 and 4).

## DISCUSSION

Comparing four populations of blue tits, our models provide support for the hypothesis that the prevalence of long‐ and short‐term RICs would differ between populations known to have fast and slow LHs, providing some support for the novel temporality in reproductive investment (TRI) hypothesis, first proposed here. As predicted, birds breeding in the less productive evergreen forests, known to have a slower LH on average, tended to contain more individuals with dominant short‐term RICs, although pronounced differences were only found between some populations. There were weak differences between males compared to females. Birds in deciduous populations showed a greater amplitude (strength) in both short‐ and long‐term RICs compared with birds in evergreen habitats, but population differences were weak for short‐term RICs. Sex differences showed males had stronger RICs compared to females when using absolute clutch size. When controlling for inter‐annual environmental variation in clutch size (mean‐centred by population‐year), we found that population differences in the prevalence of short‐ versus long‐term RICs, and the strength of long‐term RICs, persist. However, we did not detect population differences in the strength of short‐term RICs. Overall, these results show that populations that differ in their LH exhibit reproductive investment strategies over different temporal scales but that environmental variation is an important component of these differences. This provides support for the newly proposed TRI hypothesis.

### Temporality in reproductive investment strategies

Birds in evergreen populations show slow LH and a behavioural phenotype with increasing risk avoidance (Dubuc‐Messier et al., 2017) coupled with high survival probabilities (Bastianelli et al., 2021) raising the value of future reproduction. This known link between LH and survival led to our prediction that these birds would have a dominant short‐term reproductive investment strategy. Indeed, these birds appeared to pay an energetic cost of current reproduction over short periods, rapidly affecting subsequent reproductive attempts. Conversely, the potential benefit from preventing the accumulation of costs would likely not improve lifetime fitness in fast populations as their risky behavioural phenotype reduces their stochastic survival probability (Réale et al., 2010). Our results support this with deciduous populations having more prevalent dominant long‐term reproductive investment strategies.

Although the amplitude of RICs differs among populations, we suggest that both short‐ and long‐term strategies represent evolutionarily and ecologically important effects across populations. Previous work has mainly focused on modelling one or the other (e.g. Hamel et al., 2014; Nussey et al., 2008; Reed et al., 2008), but here we show that even in populations that differ in their LH, multiple RICs should be modelled. It supports the commonly held belief that the LH strategy of individuals will interact with current environment, evidenced by short‐term RICs across all populations, but reveals the surprising extent of such effects among individuals and populations.

### Amplitude of reproductive investment curves: Evidence of variation in LH strategy?

Birds with a faster LH (deciduous populations) have previously been shown to have larger and more variable clutch sizes (see Table 1 in Charmantier et al., 2016; Bastianelli et al., 2021). Long‐term RICs represent an accumulation of costs and our results showing higher amplitude for long‐term RICs overall, particularly in fast, deciduous populations, support this prediction. However, a difference in the amplitude of short‐term RICs among populations was only detected using absolute clutch size, and this relationship was weaker than when examining long‐term RICs, and when the local environment was controlled for (mean‐centred clutch size), population differences were no longer detected. This suggests that the short‐term costs of reproduction may be driven by among‐population variability in the environment.

### Amplitude of reproductive investment curves: Evidence of environmental drivers?

The comparisons we draw between absolute and mean‐centred clutch size (at the population‐year level) highlighted the importance of inter‐annual environmental variation on the amplitude of short‐term RICs. The amplitude of short‐term RICs across all populations was reduced when controlling for inter‐annual changes in the mean clutch size, and population differences no longer detected. This suggests that variation in clutch size across this temporal scale arises from environmental fluctuations, for example, in resource abundance or climatic conditions but no such effect was detected on long‐term RICs. Although our measure of environmental quality is coarse, there is substantial evidence of inter‐annual and habitat variation among the four populations used in this study, specifically with food availability within deciduous oak populations having four to five times more caterpillars than evergreen oak woodlands (Blondel et al., 2010). Future work should focus on estimates of environmental traits, preferably at small spatial scales, appropriate for individual reproductive effort, but these measures are difficult to collect in the field. However, given the complex inter‐annual variation in unmeasured environmental variables, the present method allows changes in reproductive investment to be detected without *a priori* temporal scales being defined, making it a powerful tool.

### Local and sex differences in the temporality and amplitude of resource investment curves

Differences in the amplitude of RICs aligned with differences in LH, but E‐Muro and D‐Muro often showed more similar patterns to E‐Pirio and D‐Rouviere and in some instances no detectable differences were found between these populations. Shared environmental effects across the medium scale (e.g. adjacent populations such as E‐Muro and D‐Muro) may add complexity to clutch size variation not captured by our models, and mask aspects of reproductive investment strategy (Nussey et al., 2007; van Noordwijk & De Jong, 1986; Wilson et al., 2008). A recent study showed while there is relatively high gene flow among the Corsican populations (D‐Muro, E‐Muro and E‐Pirio), there is still detectable genome wide differentiation and, therefore, the potential for local adaptation (Perrier et al., 2018) suggesting such differences in reproductive investment could have a genetic basis.

Originally, we controlled for sex differences in the models, but unexpectedly we found that male blue tits appeared to have short‐ and long‐term RICs with a higher amplitude than female blue tits and with some indication of slightly more prevalent short‐term RICs. This pattern may be driven directly by sex differences in senescence or behaviour itself, or indirectly through mate quality (Reviewed by Lemaître & Gaillard, 2017). Male provisioning rate is more responsive to increase brood size in blue tits (Griffioen et al., 2019), which could be costly to future reproduction. When controlling for inter‐annual environmental effects, the differences in the strength of RICs for males and females reduced substantially suggesting a strong environmental driver of these differences.

### Methodological constraints and future directions

A crucial characteristic of Fourier transforms is their non‐directionality. This means that an RIC between sequential reproductive attempts is not informative on when peaks and troughs in reproductive performance occur (See Supplementary material Appendix 7). Short‐term RICs may have peaks and troughs at different times depending on the environmental drivers of variation, and this could be incorporated into future models. Furthermore, RICs may not be ‘stationary’, that is, symmetrical over time. For example, environmental effects, which affect resource availability, may be irregular over time and extending the Fourier analyses to wavelet analysis (Cazelles et al., 2008) would allow investigation of whether the temporal structure of RICs is itself sensitive to conditions that are changing across time (e.g. with annual conditions).

Our study focuses on how the cost of current reproduction impacts future reproductive attempts. It does not quantify costs of current reproduction to survival, and hence the probability of reaching those future reproductive attempts. However, our results are in line with the prediction that fast birds would maintain high reproductive performance and pay the cost of an increased mortality risk, rather than a decrease in reproductive success. Here we examine birds that live until 3 years or more, which would be predicted to show senescence. Our results showing that fast populations have more individuals which fail to breed three times or more (Supplementary Material Appendix 4), and so have higher mortality, supports this hypothesis. However, including costs to survival will be an important step forward and will likely require use of non‐stationary functions (e.g. to allow a final reproductive effort of zero in the year a bird dies to be included at the end of each time series).

Although we find important drivers of differences in the temporal scale of reproductive investment, there is still a large amount of unexplained variation, evident in small parameter estimates and substantial overlap between populations. We suggest one major reason for this is the failure to account for individual level drivers of differences in LH strategies. The extension of the POL theory to include behavioural and physiological variation (Dammhahn et al., 2018; Réale et al., 2010) highlights individual level traits that may explain differences in the TRIs. There is strong support that within‐population individuals differ in a range of LH traits and we propose that individual level differences in the TRIs should be modelled in future work.

Fitting Fourier transforms to individual LHs comes with the limitation, shared with all other types of LH analyses, that birds which breed only once or twice cannot be included. Longer‐lived species should offer the possibility to both detect reproductive investment strategies over a greater set of potential time frames, or characterise among‐individual variation in dominant investment curve wavelengths more effectively. Differences in time series length and the impact on the detection of RICs of different lengths need further investigation. Although a recent study shows that birds from faster deciduous populations also have lower survival probability (Bastianelli et al., 2021), our result that these faster birds show more prevalent and stronger longer‐term RICs despite shorter lifespan and time‐series is conservative with regards to the bias that could be induced by a difference in survival across habitats. Furthermore, simulations highlight the even greater potential power to improve our understanding of reproductive investment strategies in long‐lived species, which are likely to display many different trade‐offs yet to be identified.

## CONCLUSIONS

Here, we show for the first time that differences in LH across blue tit populations correlate with the temporal scale (wavelength) and amplitude of reproductive investment strategies imposed by the cost of reproduction, supporting the TRI hypothesis. Results support the prediction that populations with a slower LH show weaker and shorter‐term RICs on average but confirm that inter‐annual environmental variation is an important driver of short‐term variation in clutch size across populations. We have demonstrated the ability to capture variation in the time scale over which reproductive costs are paid in a short‐lived species, where the average individual has only a few reproductive attempts, but perhaps the true power of this analysis will become evident when applied to long‐lived species. This modelling technique offers the potential to fully estimate reproductive investment strategies, address previous methodological limitations and hence reveal the true cost of reproduction in the wild.

## AUTHOR CONTRIBUTION

SCP and DR conceived the original idea, and developed this and the methods in conjunction with AW and JRP. SCP and JRP conducted analyses and AC, CD and CT collected field data. All authors contributed to writing the manuscript and approved the final draft for publication.

### PEER REVIEW

The peer review history for this article is available at https://publons.com/publon/10.1111/ele.13982.

## Supporting information

Supplementary Material

## Data Availability

No new data were used for this paper. All data have been shared previously. Data were obtained through SPI‐Birds https://nioo.knaw.nl/en/spi‐birds. Data and codes used in this paper can be found at: https://doi.org/10.17605/OSF.IO/Q2NRA.
