# Supplementary material for: Differences in the temporal scale of reproductive investment across the slow‐fast continuum in a passerine
Source: Ecol Lett. 2022 Mar 2;25(5):1139–51. doi: 10.1111/ele.13982 (PMC9541748; doi:10.1111/ele.13982)
Supplement: Supplementary file 1 — Supplementary Material [file ELE-25-1139-s001.docx]

# **Supplementary material: Appendix 1: Sample sizes and subsetting data**

## Table A1: The number of individuals at each population with different time series lengths D = deciduous oak; E = evergreen oak. Birds with a time series of three or more are used in the main paper analyses. Birds that breed only once or twice are examined in Supplementary material Appendix 4.

| **Time series length (breeding attempts)** | **D-Muro** | **D-Rouviere** | **E-Muro** | **E-Pirio** | **% of all individuals** | **% birds including in main analyses** |
| --- | --- | --- | --- | --- | --- | --- |
| *1* | *942* | *1032* | *253* | *790* | *61%* | *0* |
| *2* | *286* | *372* | *110* | *320* | *22%* | *0* |
| 3 | 89 | 174 | 60 | 158 | 10% | 57% |
| 4 | 40 | 82 | 27 | 86 | 5% | 28% |
| 5 | 10 | 30 | 16 | 38 | 2% | 11% |
| 6 | 4 | 5 | 4 | 9 | 0% | 3% |
| 7 | 1 | 0 | 0 | 8 | 0% | 1% |
| 8 | 0 | 0 | 0 | 4 | 0% | 0% |
| 9 | 0 | 0 | 0 | 1 | 0% | 0% |

**Supplementary material: Appendix 2: Exploring the use of Fourier modes using simulated time series**

Results using absolute clutch size showed that 15% had at least three (9% of birds in D-Muro, 12% from D-Rouviere, 19% from E-Muro and 20% from E-Pirio) and 2% had four detectable reproductive investment curves (RICs; 1% of birds from D-Muro and 4% from E-Pirio). The length of the time series strongly affected the ability to detect three or more RICs (Time series length years Mean ± SD: one RIC 3.61 ± 0.56; two RICs 3.32 ± 0.51; three RICs 5.20 ± 0.46; four RICs 7.38 ± 0.65 so it is difficult to interpret biological significance beyond the dominant and secondary RIC. Here we use simulations to examine how the time series length affects the ability to detect RICs.

**Simulation methods**

Using simulated data, we estimated the correct assignment of long and short-term RICs in relation to time series length and amplitude. We simulated time series of lengths $L=3,4,\ldots,9$. For each $L$, we simulated 100 time series with short-term RICs and 100 with long-term RICs. Each time series started with a clutch size $N_{1}$ drawn from the empirical distribution of initial clutch sizes.

Each time series with short-term RICs was constructed iteratively as follows

$$N_{k+1}={max\{N}_{k}+{c\left( -1 \right)}^{k},0\}$$

where $c$ is a random integer drawn from the empirical distribution of changes in clutch size from one year to the next (removing any situations where the clutch size does not change) and $k=2,\ldots,L$.

Each time series with long-term RICs was constructed iteratively as follows

$N_{k+1}=N_{k}+c$, if $k<4$,

$N_{k+1}={max\{N}_{k}-c,0\}$, if $k\geq4$,

where c is a random integer drawn from the empirical distribution of changes in clutch size from one year to the next (including situations where the clutch size does not change) and $k=2,\ldots,L$.

**Results**

From 1600 simulated individuals, 213 RICs were misassigned (14%). 32% of these were long-term RICs that were misassigned as short, and 68% of short-term RICs were misassigned as long-term. Further simulations and modelling is required to truly determine when and why these mismatches occur.

## Table B1: The results from simulations examining the detection of reproductive investment curves (RICs) in relation to amplitude and time series length. Simulated data were created with a given time series (3-9) and amplitude (0-3.5). Amplitudes were grouped into 0 (0-0.5); 1(0.5-1.5); 2(1.5-2.5); 3(2.5-3.5) for this summary table. This table shows when Fourier models were able to correctly assign a long (>2) or short-term (=2) RIC. The table shows % from 100 simulations per group. To allow comparisons, amplitude was rounded to the nearest whole number. If the simulations did not generate data with an amplitude-time series group, the cell is left blank. In total 92% of long-term RICs were correctly assigned and 82% of short-term RICs.

|  |  | **Long-term RIC** | | **Short-term RIC** | |
| --- | --- | --- | --- | --- | --- |
| **Timeseries** | **Amplitude** | **Incorrectly** | **Correctly** | **Incorrectly** | **Correctly** |
| **3** | **All grouped** | **6%** | **94%** | **2%** | **98%** |
|  | 0 | 23% | 77% | 0% | 100% |
|  | 1 | 0% | 100% | 0% | 100% |
|  | 2 | 0% | 100% | 18% | 82% |
|  | 3 | 0% | 100% | 0% | 100% |
| **4** | **All grouped** | **35%** | **65%** | **18%** | **82%** |
|  | 0 | 75% | 25% |  |  |
|  | 1 | 27% | 73% | 15% | 85% |
|  | 2 | 41% | 59% | 11% | 89% |
|  | 3 |  | 100% |  |  |
| **5** | **All grouped** | **17%** | **83%** | **9%** | **91%** |
|  | 0 | 35% | 65% | 0% | 100% |
|  | 1 | 17% | 83% | 8% | 92% |
|  | 2 | 0% | 100% | 33% | 67% |
|  | 3 | 0% | 100% |  |  |
| **6** | **All grouped** | **2%** | **98%** | **17%** | **83%** |
|  | 0 | 20% | 80% |  |  |
|  | 1 | 0% | 99% | 12% | 88% |
|  | 2 | 0% | 100% | 60% | 40% |
|  | 3 | 0% | 100% |  |  |
| **7** | **All grouped** | **6%** | **94%** | **17%** | **83%** |
|  | 0 | 7% | 93% | 0% | 100% |
|  | 1 | 7% | 93% | 13% | 87% |
|  | 2 | 0% | 100% | 56% | 44% |
|  | 3 | 0% | 100% |  |  |
| **8** | **All grouped** | 0% | **100%** | **24%** | **76%** |
|  | 0 | 0% | 100% |  |  |
|  | 1 | 0% | 100% | 18% | 82% |
|  | 2 | 0% | 100% | 60% | 40% |
|  | 3 | 0% | 100% |  |  |
| **9** | **All grouped** | **2%** | **98%** | **35%** | **65%** |
|  | 0 | 33% | 67% |  | 100% |
|  | 1 | 0% | 100% | 30% | 70% |
|  | 2 | 0% | 100% | 88% | 12% |
|  | 3 | 0% | 100% |  |  |
|  |  |  |  |  |  |

#
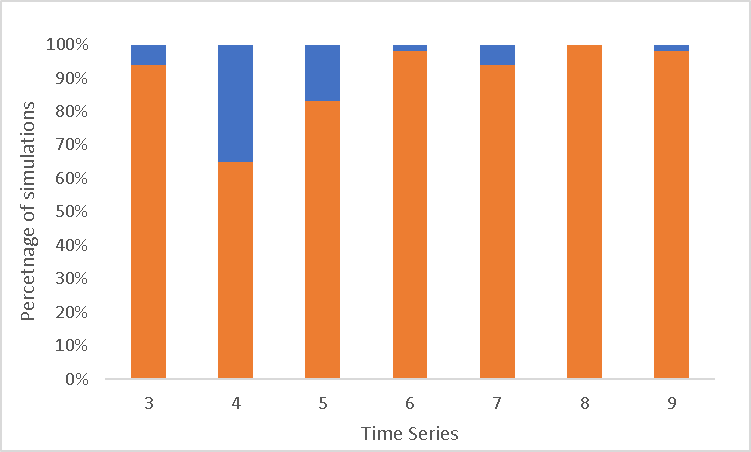

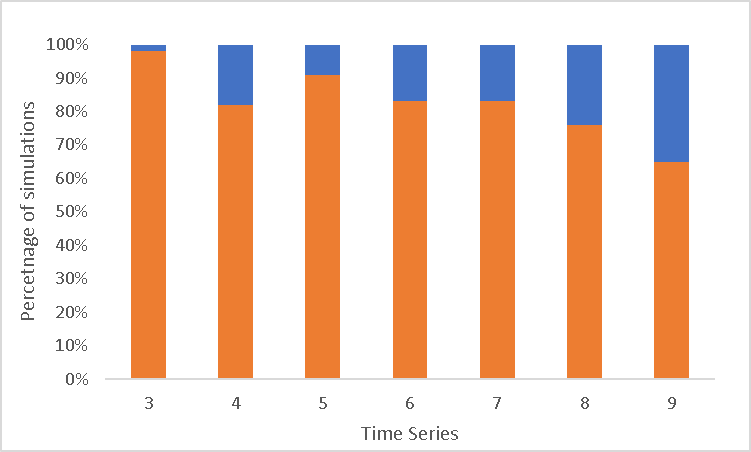


a)

b)

Figure B1: The results from simulations examining the detection of reproductive investment curves in relation to amplitude and time series length. Simulated data were created with a given time series (3-9) and the average across all amplitudes are shown here (See Table B1 for full details. This figure shows when Fourier models were able to correctly assign a long (>2; Figure B1a) or short-term (=2; Figure B1b) RIC. Orange bars = correctly assigned and blue = incorrectly assigned.

**Supplementary material: Appendix 3: Dominant and secondary wavelengths and associated amplitudes**

## Table C1: The percentage of individuals in each population exhibiting dominant reproductive investment curves (RICs; i.e. the RIC with the highest amplitude) of differing wavelengths, using absolute and mean-centred clutch size. This is for individual birds that breed three or more times. D = deciduous oak; E = evergreen oak.

|  | N | % | N | % | N | % | N | % | N | % | N | % | N | % | N | | % |
| --- | --- | --- | --- | --- | --- | --- | --- | --- | --- | --- | --- | --- | --- | --- | --- | --- | --- |
|  | D-Muro | | | | D-Rouviere | | | | E-Muro | | | | E-Pirio | | | | |
| Wavelength of Fourier mode | Absolute clutch size | | Mean-centred clutch size | | Absolute clutch size | | Mean-centred clutch size | | Absolute clutch size | | Mean-centred clutch size | | Absolute clutch size | | | Mean-centred clutch size | |
| 2 | 57 | 41% | 53 | 37% | 108 | 41% | 122 | 43% | 41 | 41% | 49 | 46% | 138 | 49% | 140 | | 46% |
| 2.666667 |  | 0% |  | 0% |  | 0% |  | 0% |  | 0% |  | 0% | 2 | 1% | 1 | | 0% |
| 2.8 | 1 | 1% | 1 | 1% |  | 0% |  | 0% |  | 0% |  | 0% | 3 | 1% | 2 | | 1% |
| 3 | 2 | 1% | 2 | 1% | 3 | 1% | 1 | 0% | 3 | 3% | 2 | 2% | 6 | 2% | 5 | | 2% |
| 3.333333 | 5 | 4% | 4 | 3% | 14 | 5% | 15 | 6% | 6 | 6% | 6 | 6% | 18 | 6% | 23 | | 8% |
| 4 | 16 | 12% | 26 | 18% | 45 | 16% | 55 | 17% | 12 | 12% | 18 | 17% | 30 | 11% | 54 | | 18% |
| 4.666667 |  | 0% |  | 0% |  | 0% |  | 0% |  | 0% |  | 0% | 2 | 1% | 2 | | 1% |
| 6 | 54 | 39% | 54 | 38% | 92 | 34% | 90 | 33% | 32 | 32% | 28 | 26% | 74 | 26% | 68 | | 22% |
| 8 |  | 0% |  | 0% |  | 0% |  | 0% |  | 0% |  | 0% |  | 0% | 2 | | 1% |
| 10 | 4 | 3% | 4 | 3% | 7 | 3% | 8 | 3% | 5 | 5% | 4 | 4% | 5 | 2% | 5 | | 1% |
| 14 |  | 0% |  | 0% |  | 0% |  | 0% |  | 0% |  | 0% | 1 | 0% | 1 | | 0% |
| 18 |  | 0% |  | 0% |  | 0% |  | 0% |  | 0% |  | 0% | 1 | 0% | 1 | | 0% |

##
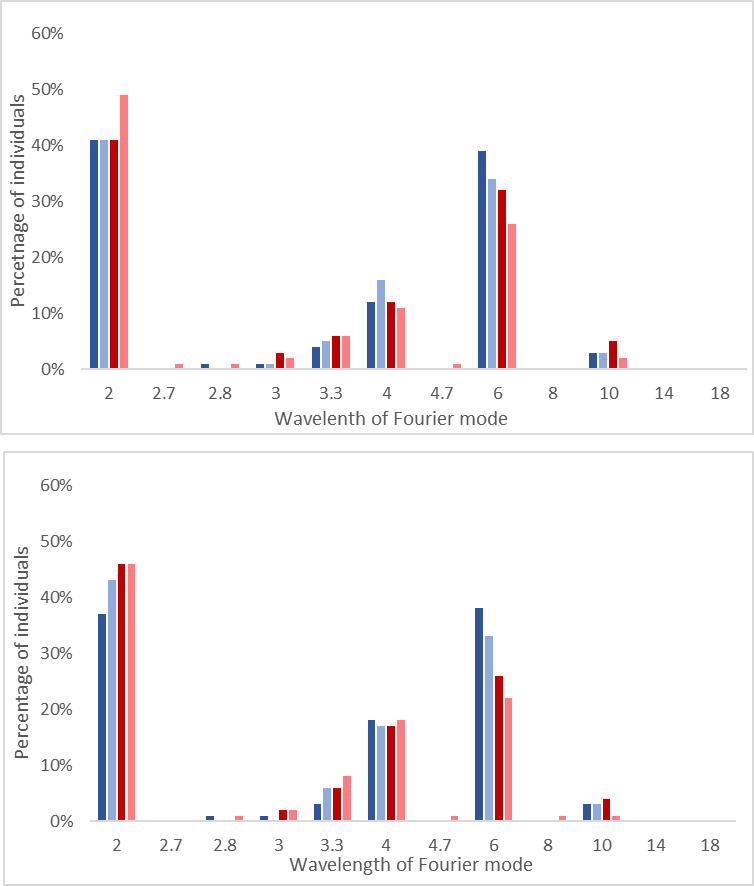


a)

b)

## Figure C1: The percentage of individuals in each population exhibiting dominant reproductive investment curves (RICs; i.e. the RIC with the highest amplitude) of differing wavelengths, using absolute clutch size (Figure C1a) and mean-centred clutch size (Figure C1b). This is for individual birds that breed three or more times. D-Muro – dark blue; D-Rouviere – pale blue; E-Muro – dark red; E-Pirio – pale red.

## Table C2: The percentage of individuals in each population exhibiting secondary reproductive investment curves (RICs; i.e. the RIC with the second-highest amplitude) of differing wavelengths. This is for individual birds that breed three or more times. D = deciduous oak; E = evergreen oak.

|  | N | % | N | % | N | % | N | % | N | % | N | % | N | % | N | % |
| --- | --- | --- | --- | --- | --- | --- | --- | --- | --- | --- | --- | --- | --- | --- | --- | --- |
|  | D-Muro | | | | D-Rouviere | | | | E-Muro | | | | E-Pirio | | | |
|  | Absolute clutch size | | Mean-centred clutch size | | Absolute clutch size | | Mean-centred clutch size | | Absolute clutch size | | Mean-centred clutch size | | Absolute clutch size | | Mean-centred clutch size | |
| 2 | 55 | 53% | 83 | 58% | 109 | 53% | 152 | 52% | 39 | 47% | 48 | 45% | 93 | 41% | 132 | 43% |
| 2.57143 |  | 0% |  | 0% |  | 0% |  | 0% |  | 0% |  | 0% | 1 | 0% |  | 1% |
| 2.666667 |  | 0% |  | 0% |  | 0% |  | 0% |  | 0% |  | 0% | 1 | 0% |  | 1% |
| 2.8 |  | 0% |  | 0% |  | 0% |  | 0% |  | 0% |  | 0% | 2 | 1% | 3 | 3% |
| 3 | 1 | 1% | 1 | 1% | 2 | 1% | 2 | 1% | 1 | 1% | 1 | 1% | 2 | 1% | 4 | 0% |
| 3.333333 | 5 | 4% | 6 | 4% | 9 | 3% | 7 | 2% | 8 | 8% | 7 | 7% | 15 | 5% | 10 | 12% |
| 3.6 |  |  |  |  |  |  |  |  |  |  |  |  |  | 0% | 1 | 0% |
| 4 | 24 | 17% | 14 | 10% | 35 | 13% | 27 | 9% | 13 | 13% | 9 | 8% | 47 | 17% | 35 | 1% |
| 4.666667 | 1 | 1% |  | 0% |  | 0% |  | 0% |  | 24% |  | 0% | 3 | 1% | 3 | 31% |
| 6 | 33 | 24% | 37 | 26% | 66 | 24% | 88 | 30% | 23 | 6% | 36 | 34% | 73 | 27% | 94 | 7% |
| 8 |  | 0% |  | 0% |  | 0% |  | 0% |  | 0% | 6 | 6% | 2 | 1% | 21 | 0% |
| 10 | 1 | 1% | 2 | 1% | 13 | 5% | 15 | 5% | 6 | 0% |  | 0% | 14 | 5% | 1 | 0% |
| 14 |  |  | 1 | 1% |  |  |  |  |  |  |  |  | 1 | 0% |  | 0% |
|  |  |  |  |  |  |  |  |  |  |  |  |  |  |  |  |  |
|  |  |  |  |  |  |  |  |  |  |  |  |  |  |  |  |  |
|  |  |  |  |  |  |  |  |  |  |  |  |  |  |  |  |  |
|  |  |  |  |  |  |  |  |  |  |  |  |  |  |  |  |  |
|  |  |  |  |  |  |  |  |  |  |  |  |  |  |  |  |  |
|  |  |  |  |  |  |  |  |  |  |  |  |  |  |  |  |  |
|  |  |  |  |  |  |  |  |  |  |  |  |  |  |  |  |  |
|  |  |  |  |  |  |  |  |  |  |  |  |  |  |  |  |  |

##
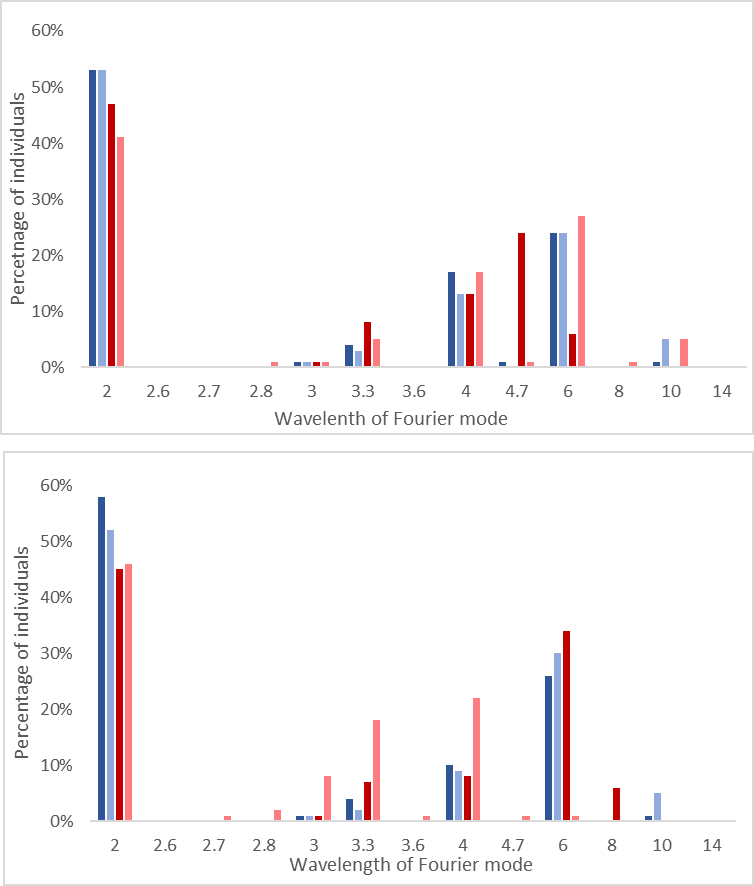


b)

a)

## Figure C2: The percentage of individuals in each population exhibiting secondary reproductive investment curves (RICs; i.e. the RIC with the second-highest amplitude) of differing wavelengths, using absolute clutch size (Figure C2a) and mean-centred clutch size (Figure C2b). This is for individual birds that breed three or more times. D-Muro – dark blue; D-Rouviere – pale blue; E-Muro – dark red; E-Pirio – pale red.

##
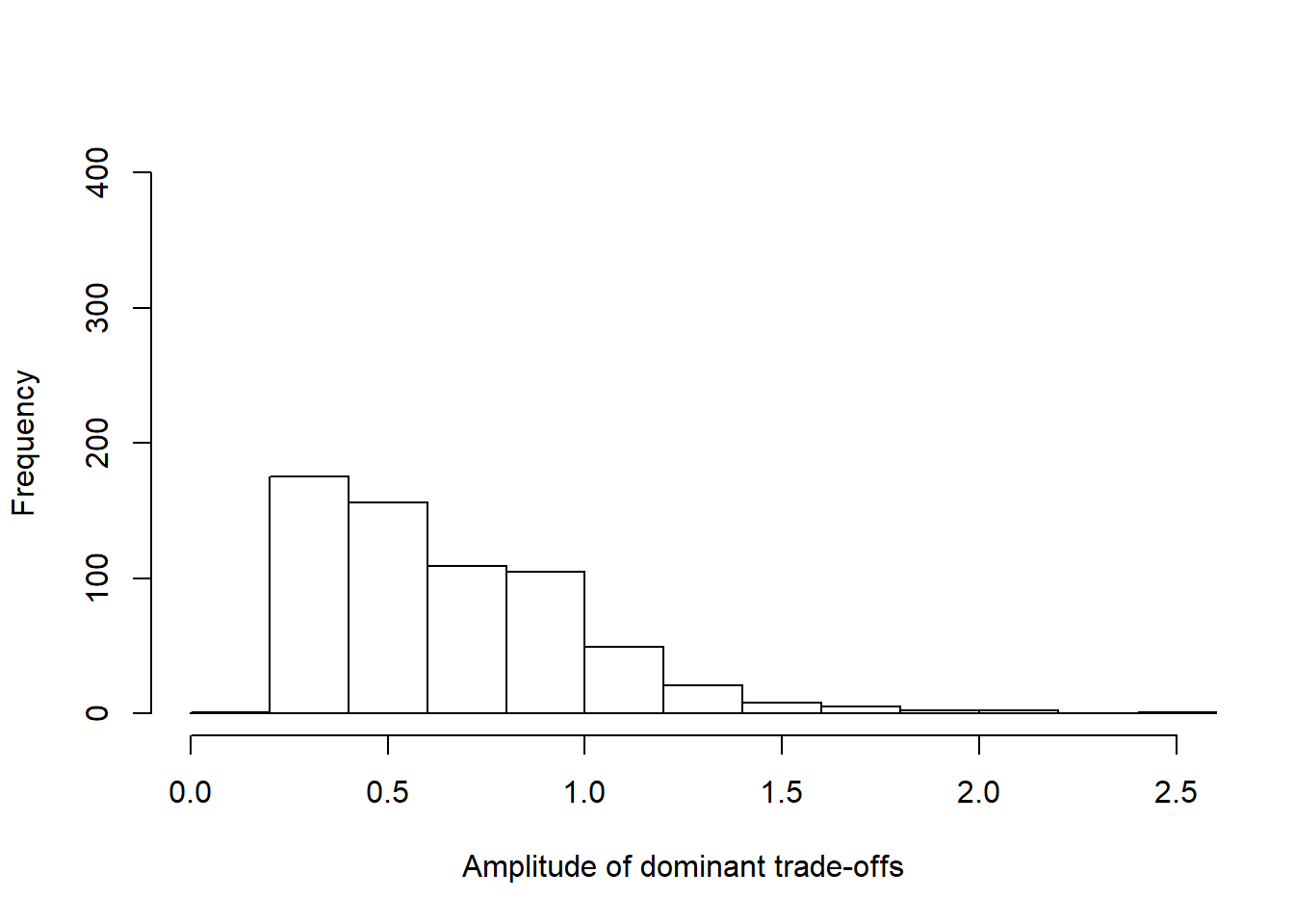

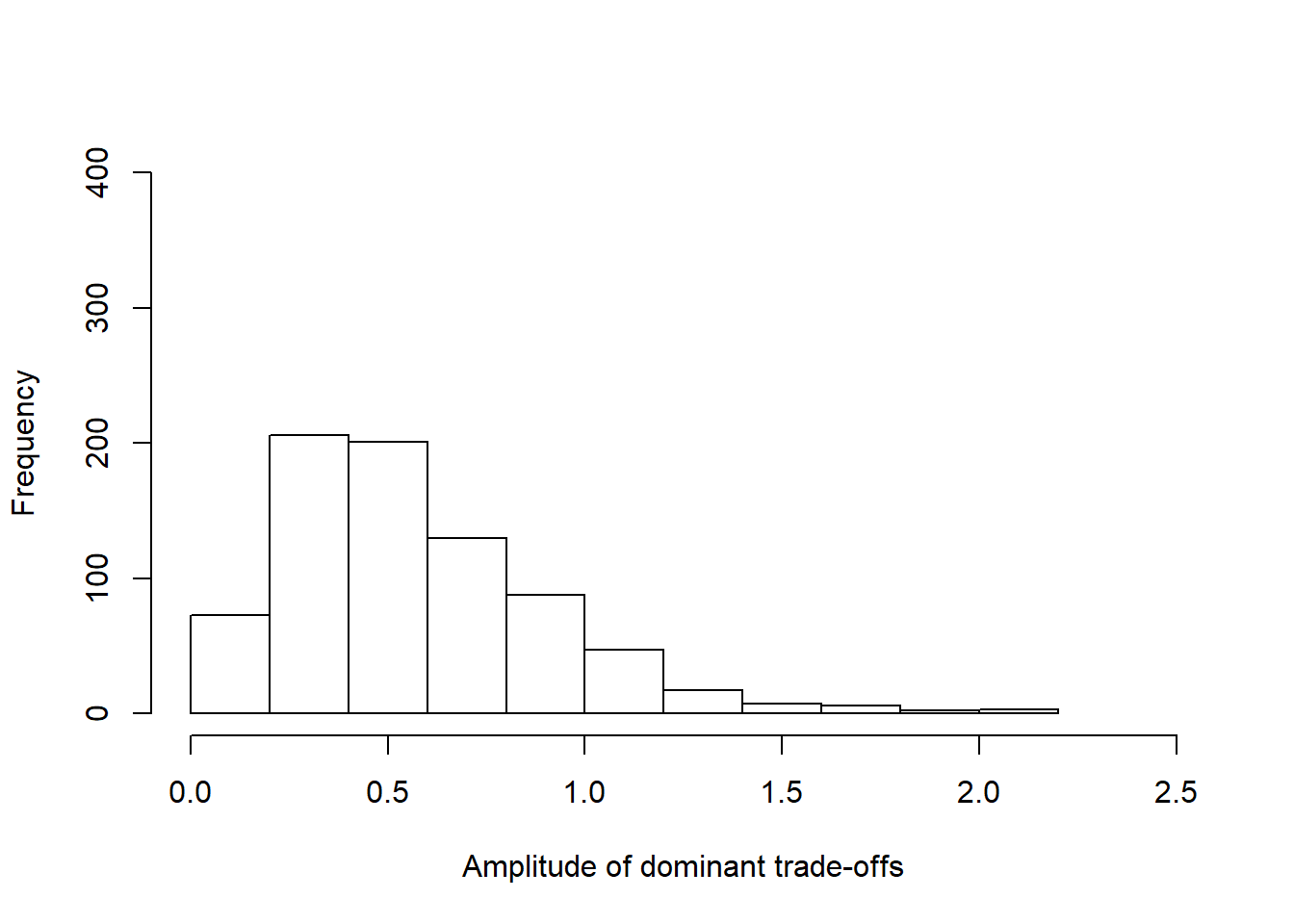


e)

a)

##
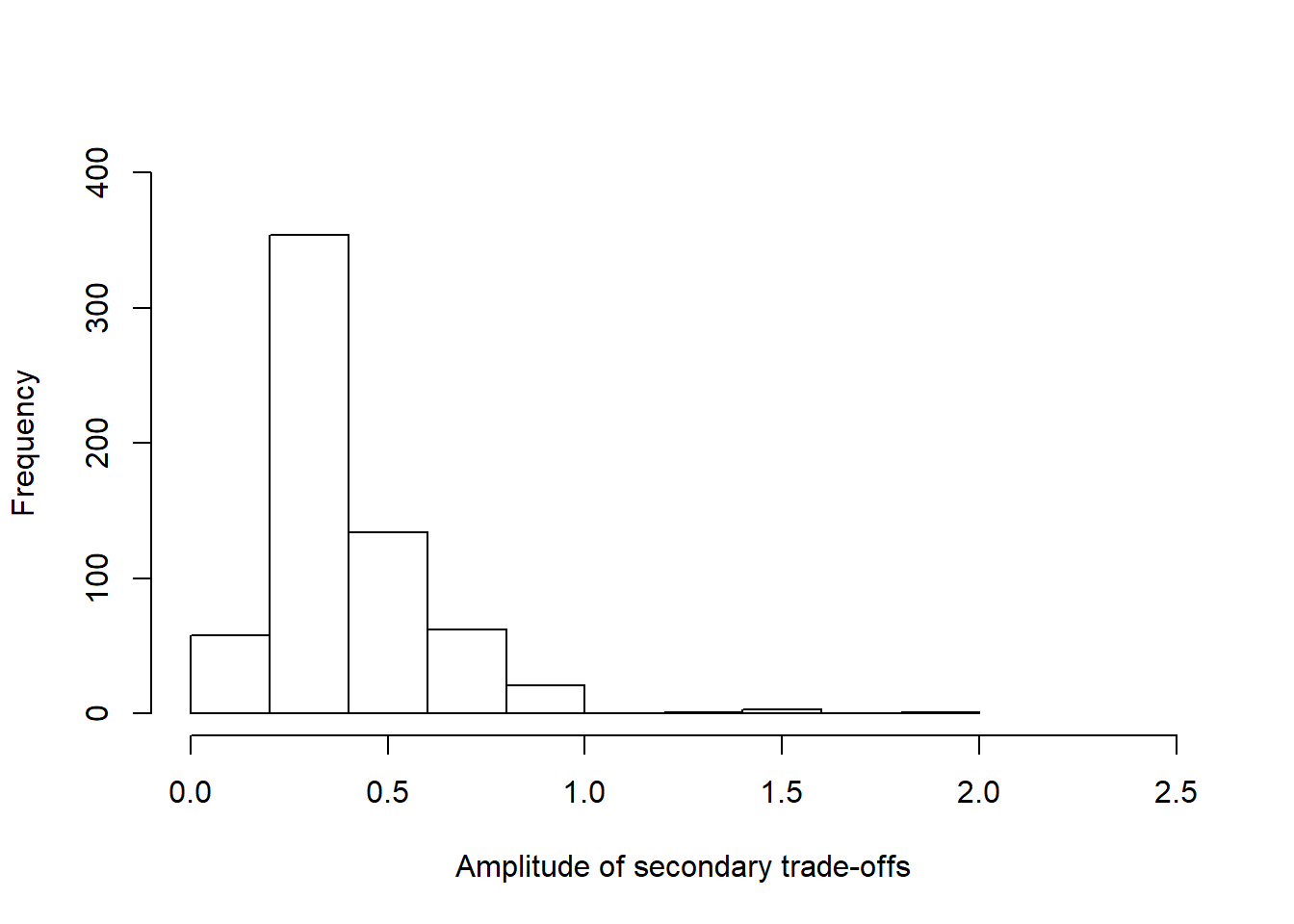

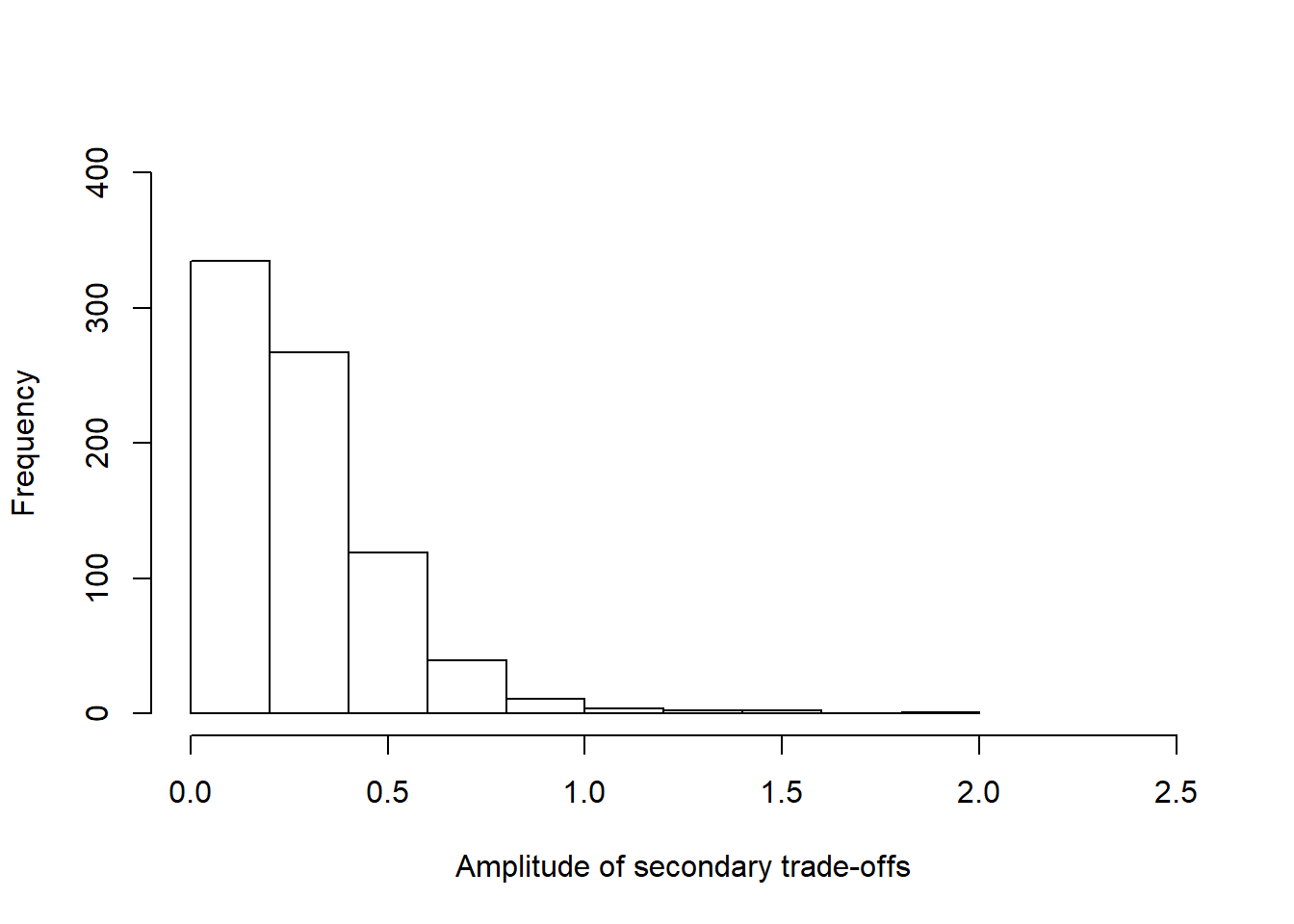

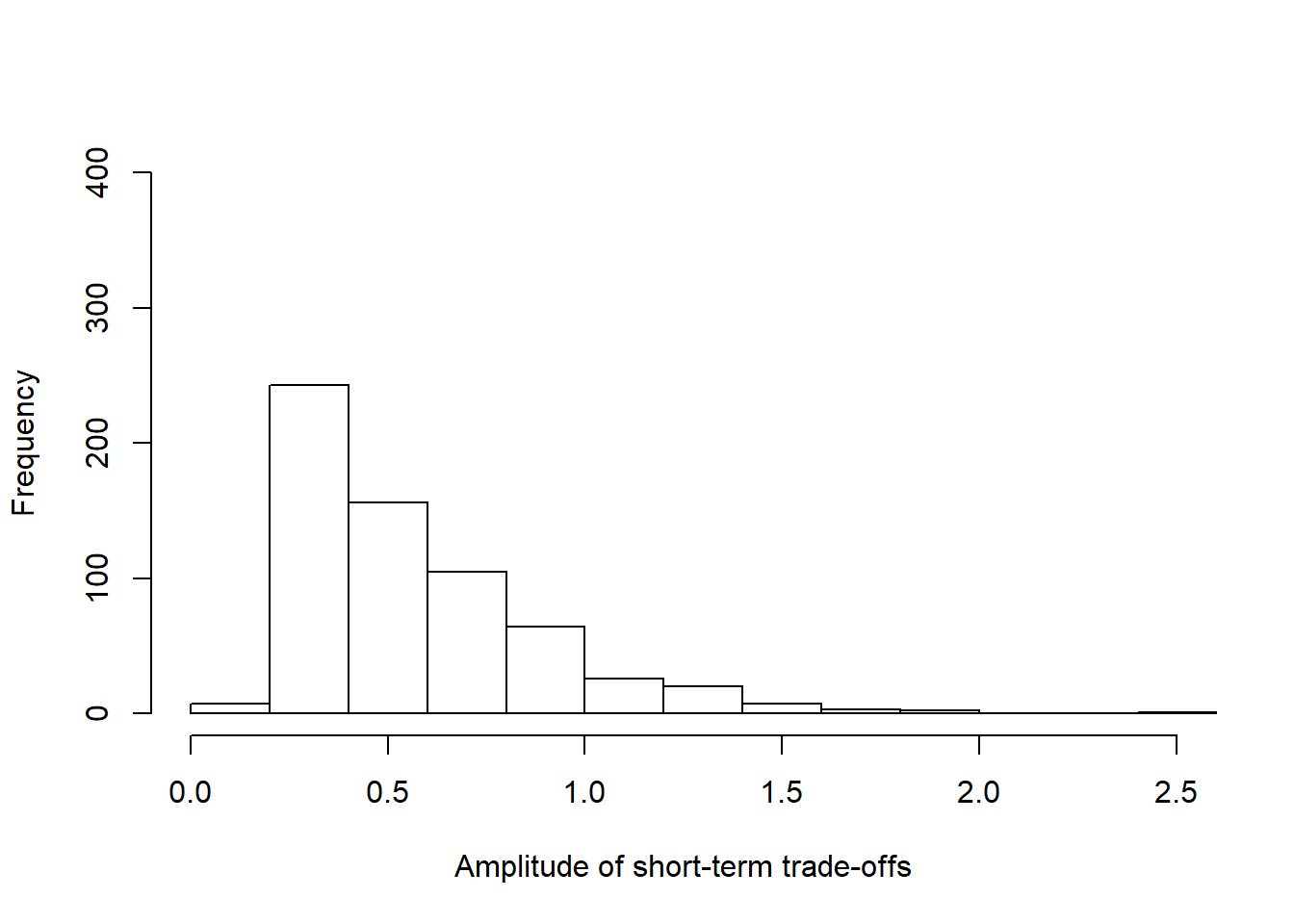

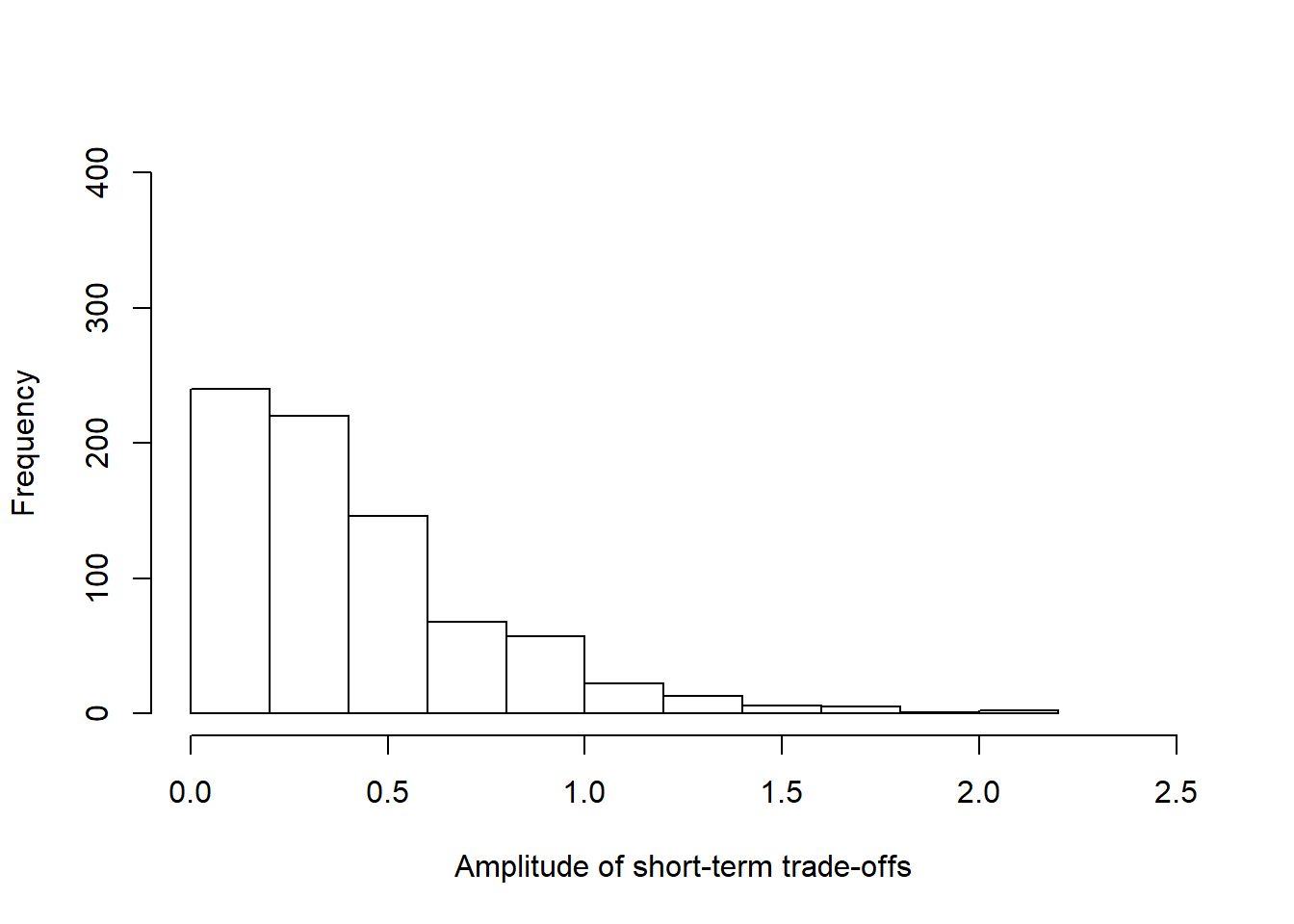


g)

c)

f)

b)


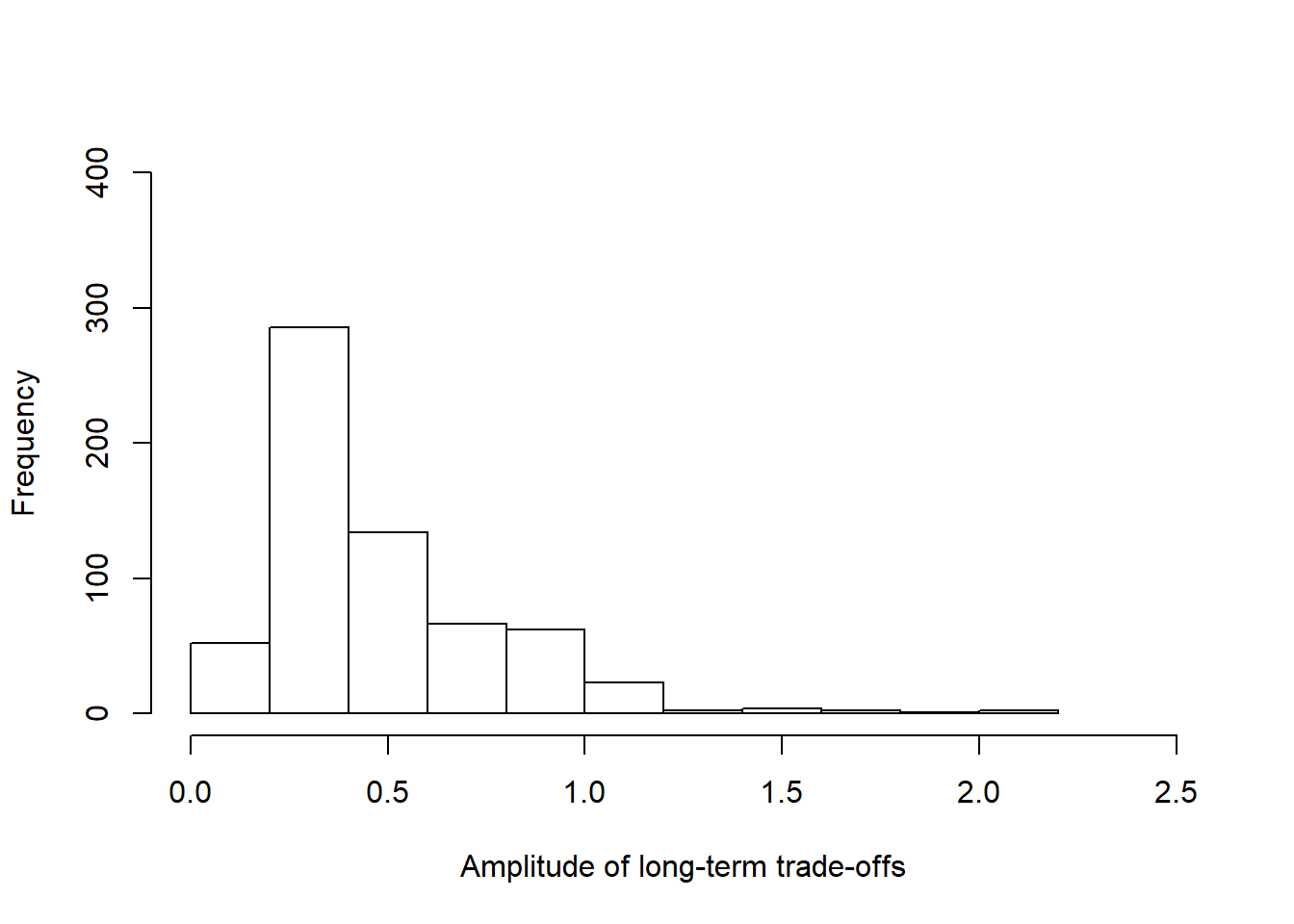

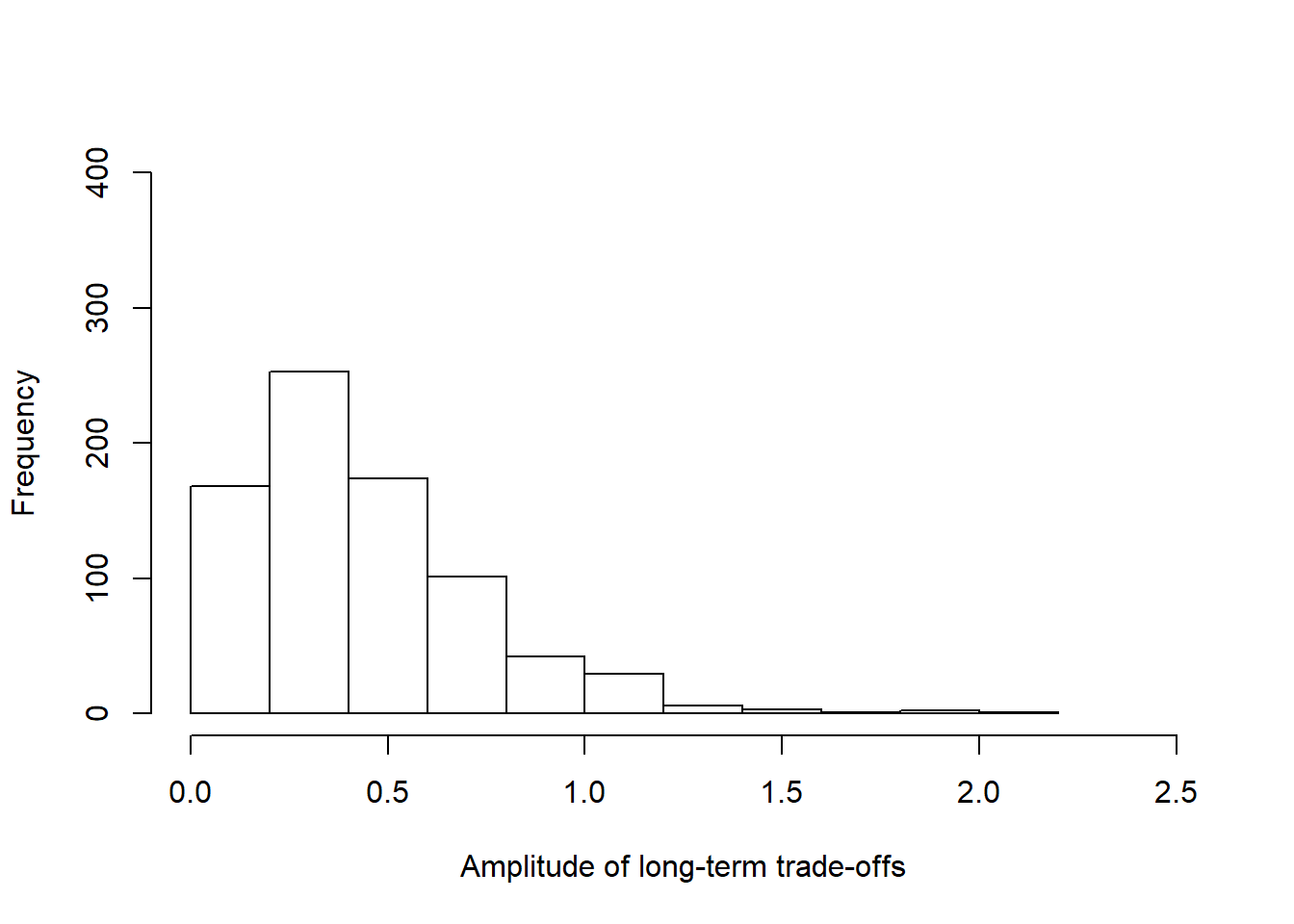


h)

d)

## Figure C3: Distribution of amplitudes across all four populations combined using absolute clutch size (a-d) and mean-centred clutch size (e-h). a,e) a histogram of amplitudes for the dominant reproductive investment curve (RIC), defined as the wavelength with the highest amplitude, for each individual b,f) a histogram of amplitudes for the secondary RIC, defined as the wavelength with the second-highest amplitude, for each individual c,g) a histogram of amplitudes for the short-term RIC, defined as RICs with a wavelength = 2, for each individual d,h) a histogram of amplitudes for the long-term RIC, defined as RICs with a wavelength more than 2, for each individual.

# **Supplementary material: Appendix 4: Birds that breed less than three times**

We conducted an analysis combining all data from all four populations to compare the proportion of individuals among the populations which reproduced for only one or two years only versus three years or more. A binomial generalised linear mixed model was fitted with two categories: 0 – a bird that reproduced once or twice; 1 – a bird reproduced three times or more. Population, sex and mean individual clutch size (indicative of individual quality differences) were fitted as fixed effects and birth year, to account for cohort effects, as a random intercept. Data from birds that had their first breeding attempt in the last two years were excluded for this analysis (2016 and 2017). Full models are shown in Table D1.

D-Muro had the smallest proportion of individuals breeding three times or more, and E-Pirio and E-Muro the largest (Table D1; Figure D1), and males had a lower probability of breeding three times or more compared to females (Table D1).

Table D1: Results from the generalised linear mixed model examining the drivers of whether breeding blue tits live until 3 years old. Binary models where 0 = bred for only one or two years and 1 = bred for 3 years or more (N = 4951). Birth Year was fitted as a random intercept in all models (σ^2^ = 0.090). a) Full model results and in bold, the model with the minimum AIC, and all models with a delta AIC<2 which were selected. b) Best fitting model(s) are shown with (averaged) parameter estimates and model weighting. Variables included in each model are marked with + and empty cells where variables were dropped.

a)

| **Intercept** | **Mean Individual Clutch Size** | **Population** | **Sex** | **df** | **logLik** | **AICc** | **delta** | **weight** |
| --- | --- | --- | --- | --- | --- | --- | --- | --- |
| **-2.02** | **0.11** | **+** | **+** | **7** | **-2207.20** | **4428.42** | **0.00** | **0.71** |
| **-2.01** |  | **+** | **+** | **6** | **-2209.15** | **4430.31** | **1.89** | **0.28** |
| -2.14 | 0.11 | + |  | 6 | -2213.04 | 4438.09 | 9.67 | 0.01 |
| -2.13 |  | + |  | 5 | -2214.88 | 4439.78 | 11.36 | 0.00 |
| -1.44 |  |  | + | 3 | -2243.20 | 4492.40 | 63.98 | 0.00 |
| -1.45 | -0.05 |  | + | 4 | -2242.48 | 4492.97 | 64.55 | 0.00 |
| -1.56 |  |  |  | 2 | -2248.40 | 4500.81 | 72.39 | 0.00 |
| -1.56 | -0.05 |  |  | 3 | -2247.71 | 4501.42 | 73.00 | 0.00 |

## b)

| **Parameter** | **Parameter Estimate** | | **Standard Error** | |
| --- | --- | --- | --- | --- |
| Intercept^1^ | | -2.02 | | 0.11 |
| D-Rouviere | | 0.49 | | 0.12 |
| E-Muro | | 0.97 | | 0.15 |
| E-Pirio | | 0.90 | | 0.13 |
| Mean Individual Clutch Size | | 0.08 | | 0.07 |
| Sex - Male | | -0.26 | | 0.08 |

^1^ D-Muro is the population of reference and females the sex reference.


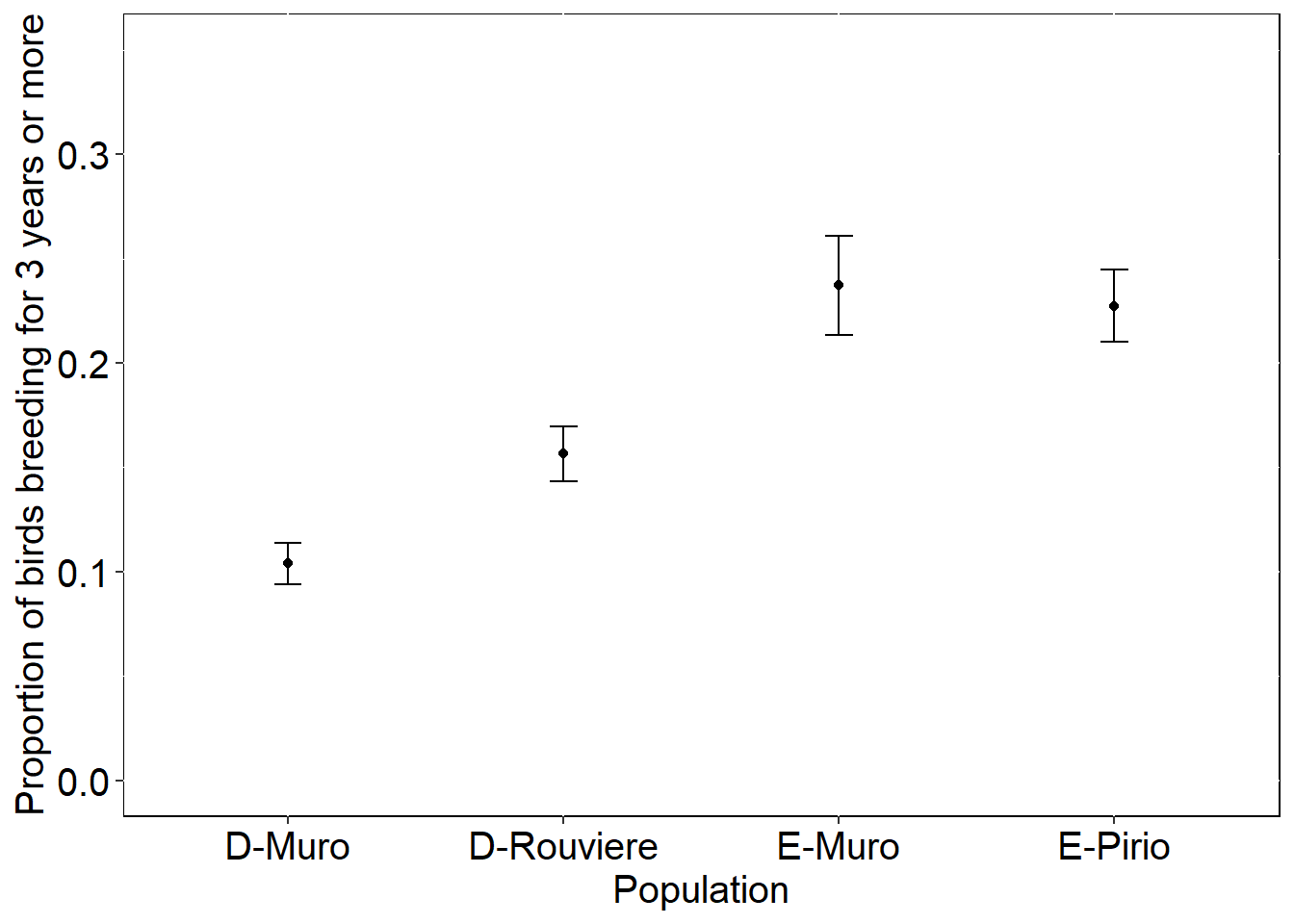


## Figure D1: Life history reproductive investment curves (RICs) among four blue tit populations, living in different habitats (D = deciduous oak; E = evergreen oak). Differences in the proportion of birds which have recruited to the breeding population, which then breed three times or more between the different populations.

# **Supplementary material: Appendix 5: Categorising short- and long-term reproductive investment curves**

Reproductive investment curves (RICs) were grouped into short- and long for several reasons:

- There is a clear peak in the number of individuals with a dominant wavelength of 2, 4 and 6 years.
- In order to compare the strength of long- and short-term RICs a categorical classification was needed
- Modelling a three-level RIC is statistically more challenging and there is a strong skew towards 2 and 6.
- In the main paper, we present results using 2 years and >2 years. We selected this as it is the most biologically plausible RIC pattern. In Appendix 6 we use a cut off of <4 years and 4 years + as statistically, this appears to be another cut off shown by the data.


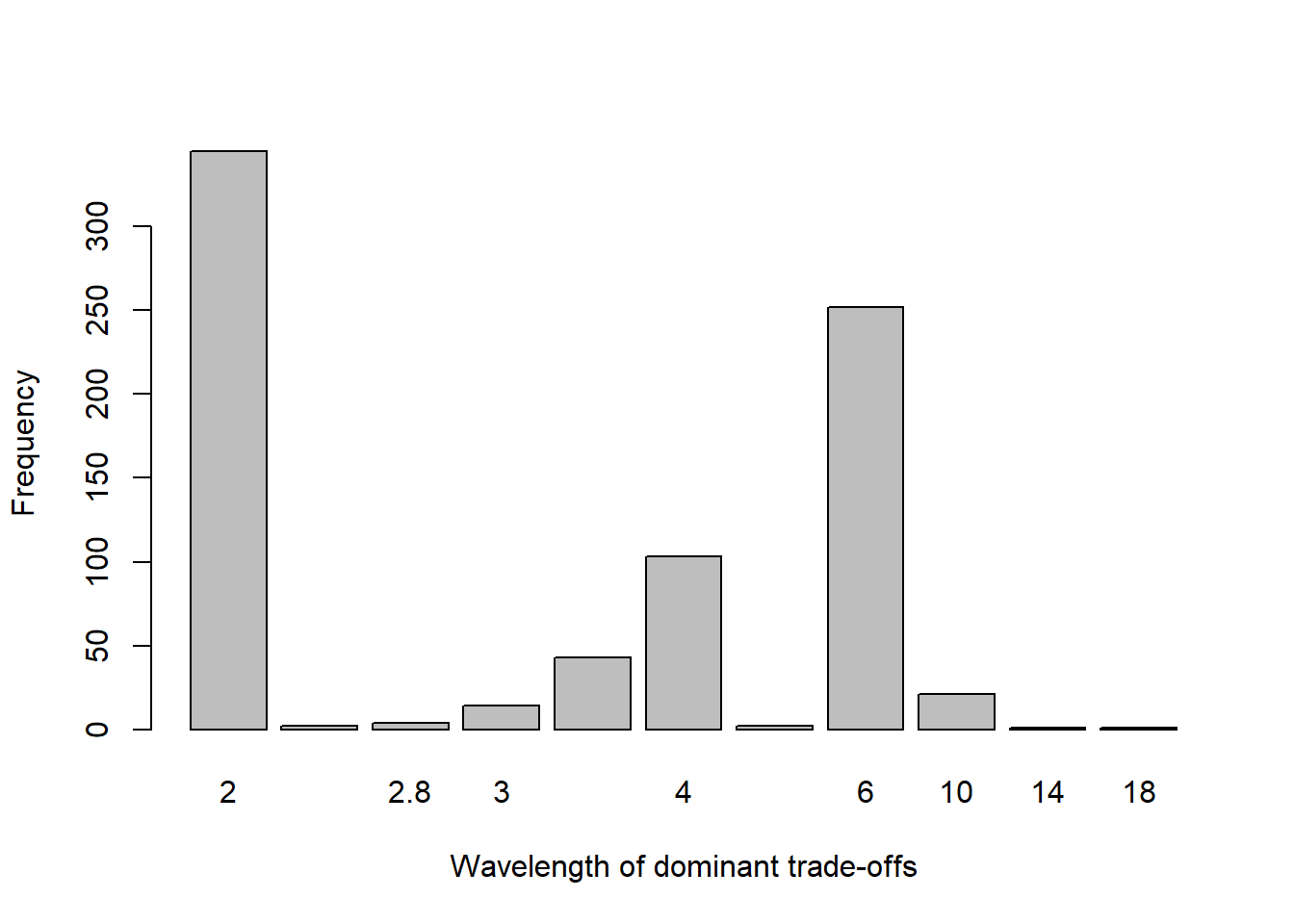

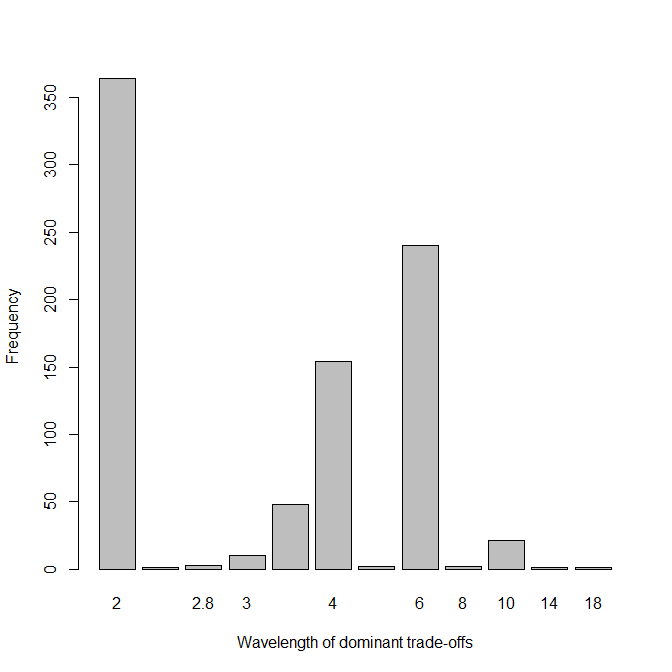


b)

a)

## Figure E1: The distribution of dominant wavelengths across the population a) Wavelengths calculated using absolute clutch size data b) Wavelengths calculated using mean-centred clutch size data

**Supplementary material: Appendix 6: Results from the main text using a cut off for short- and long-term reproductive investmenet curves of < 4 years and 4 years +**

Table F1: Results from the generalised linear mixed model examining the drivers of whether breeding blue tits show dominant short- or long-term reproductive investment curves (RICs). Binary models where 0 = dominant RIC is short-term (wavelength of less than 4 years) and 1 = dominant RIC is long-term (wavelength of 4 years+). Absolute clutch size N = 787; Mean-centred clutch size N = 846. Birth Year was included in all models (Absolute clutch size: σ^2^ = 0.000; Mean-centred clutch size σ^2^ = 0.000). a) Full model results and in bold, the model with the minimum AIC, and all models with a delta AIC<2 which were selected. b) Best fitting model(s) are shown with (averaged) parameter estimates and model weighting. Variables included in each model are marked with + and empty cells where variables were dropped. Results are shown using absolute clutch size and mean-centred clutch size.

## a)

| **Intercept** | **Mean Individual Clutch Size** | **Population** | | **Sex** | **df** | **logLik** | **AICc** | **delta** | **weight** |
| --- | --- | --- | --- | --- | --- | --- | --- | --- | --- |
| ***Absolute clutch size*** | | | | | | | | | |
| **0.18** | **-0.26** | **+** | |  | **6** | **-537.49** | **1087.10** | **0.00** | **0.46** |
| **0.13** |  | **+** | |  | **5** | **-539.30** | **1088.70** | **1.59** | **0.21** |
| **0.14** | **-0.26** | **+** | | **+** | **7** | **-537.29** | **1088.70** | **1.65** | **0.20** |
| 0.09 |  | + | | + | 6 | -539.14 | 1090.40 | 3.31 | 0.09 |
| -0.07 | 0.11 |  | |  | 3 | -543.88 | 1093.80 | 6.71 | 0.02 |
| -0.07 |  |  | |  | 2 | -545.04 | 1094.10 | 7.02 | 0.01 |
| -0.11 | 0.11 |  | | + | 4 | -543.69 | 1095.40 | 8.35 | 0.01 |
| -0.11 |  |  | | + | 3 | -544.84 | 1095.70 | 8.63 | 0.01 |
| ***Mean-centred clutch size*** | | | | | | | | | |
| **0.34** |  | **+** |  | | **5** | **-581.40** | **1172.90** | **0.00** | **0.36** |
| **0.39** |  | **+** | **+** | | **6** | **-581.14** | **1174.40** | **1.52** | **0.17** |
| **-0.01** | **0.14** |  |  | | **3** | **-584.29** | **1174.60** | **1.75** | **0.15** |
| 0.33 | 0.01 | + |  | | 6 | -581.39 | 1174.90 | 2.02 | 0.13 |
| 0.02 | 0.14 |  | + | | 4 | -584.09 | 1176.20 | 3.37 | 0.07 |
| 0.38 | 0.01 | + | + | | 7 | -581.14 | 1176.40 | 3.54 | 0.06 |
| -0.01 |  |  |  | | 2 | -586.38 | 1176.80 | 3.91 | 0.05 |
|  |  |  |  | |  |  |  |  |  |

b)

| **Parameter** | **Parameter Estimate** | | **Standard Error** | | **Parameter Estimate** | **Standard Error** |
| --- | --- | --- | --- | --- | --- | --- |
|  | | **Absolute clutch size** | | | **Mean-centred clutch size** | |
| Intercept^1^ | | 0.16 | | 0.18 | 0.27 | 0.22 |
| D-Rouviere | | 0.18 | | 0.25 | -0.18 | 0.21 |
| E-Muro | | -0.31 | | 0.29 | -0.36 | 0.30 |
| E-Pirio | | -0.75 | | 0.28 | -0.46 | 0.30 |
| Mean Individual Clutch Size | | 0.02 | | 0.08 | 0.03 | 0.07 |
| Sex - Male | | -0.21 | | 0.16 | -0.02 | 0.08 |

^1^ D-Muro is the population of reference and females the sex reference

Table F2: Results from the general linear model examining the change in clutch size associated with short-term (wavelength < 4) reproductive investment curves (RICs) in individuals, based on amplitude. Models of absolute clutch size were log-transformed and mean-centred clutch size were square root transformed. Absolute clutch size N = 652; Mean-centred clutch size N = 807. Birth Year was included in all models (Absolute clutch size: σ^2^ = 0.000; Mean-centred clutch size σ^2^ = 0.022). a) Full model results and in bold, the model with the minimum AIC, and all models with a delta AIC<2 which were selected. b) Best fitting model(s) are shown with (averaged) parameter estimates and model weighting. Variables included in each model are marked with + and empty cells where variables were dropped. Results are shown using absolute clutch size and mean-centred clutch size.

a)

| **Intercept** | **Mean Individual Clutch Size** | **Population** | **Sex** | **df** | **logLik** | **AICc** | **delta** | **weight** |
| --- | --- | --- | --- | --- | --- | --- | --- | --- |
| ***Absolute clutch size*** | | | | | | | | |
| **-0.89** | **0.08** |  | **+** | **5** | **-578.59** | **1167.3** | **0** | **0.62** |
| **-0.85** | **0.08** | **+** | **+** | **8** | **-576.4** | **1169.0** | **1.74** | **0.26** |
| -0.83 |  | + | + | 7 | -578.27 | 1170.7 | 3.44 | 0.11 |
| -0.89 |  |  | + | 4 | -584.67 | 1177.4 | 10.13 | 0 |
| -0.82 | 0.08 |  |  | 4 | -584.69 | 1177.4 | 10.17 | 0 |
| -0.78 | 0.09 | + |  | 7 | -582.63 | 1179.4 | 12.15 | 0 |
| -0.76 |  | + |  | 6 | -584.59 | 1181.3 | 14.04 | 0 |
| -0.82 |  |  |  | 3 | -590.57 | 1187.2 | 19.9 | 0 |
| ***Mean-centred clutch size*** | | | | | | | | |
| **0.56** |  |  | **+** | **4** | **-38.1** | **84.2** | **0** | **0.61** |
| **0.56** | **0.01** |  | **+** | **5** | **-37.74** | **85.6** | **1.32** | **0.31** |
| 0.57 |  | + | + | 7 | -37.63 | 89.4 | 5.15 | 0.05 |
| 0.57 | 0.01 | + | + | 8 | -37.5 | 91.2 | 6.94 | 0.02 |
| 0.59 |  |  |  | 3 | -43.36 | 92.7 | 8.5 | 0.01 |
| 0.59 | 0.01 |  |  | 4 | -43 | 94 | 9.81 | 0.01 |
| 0.6 |  | + |  | 6 | -42.9 | 97.9 | 13.67 | 0 |
| 0.6 | 0.01 | + |  | 7 | -42.74 | 99.6 | 15.38 | 0 |

b)

| **Parameter** | **Parameter Estimate** | | **Standard Error** | | **Parameter Estimate** | **Standard Error** |
| --- | --- | --- | --- | --- | --- | --- |
|  | | **Absolute clutch size** | | | **Mean-centred clutch size** | |
| Intercept^1^ | | -0.88 | | 0.05 | 0.56 | 0.01 |
| D-Rouviere | | -0.02 | | 0.05 |  |  |
| E-Muro | | -0.05 | | 0.09 |  |  |
| E-Pirio | | 0.00 | | 0.05 |  |  |
| Mean Individual Clutch Size | | 0.08 | | 0.03 | 0.00 | 0.01 |
| Sex - Male | | 0.16 | | 0.05 | 0.06 | 0.02 |

^1^D-Muro is the population of reference and females the sex reference.

Table F3: Results from the general linear model examining the change in clutch size associated with long-term (wavelength of 4 years or more) reproductive investment curves (RICs) in individuals, based on amplitude. Models of absolute clutch size were log-transformed and mean-centred clutch size were square root transformed. Absolute clutch size N = 652; Mean-centred clutch size N = 807. Birth Year was included in all models (Absolute clutch size: σ^2^ = 0.003; Mean-centred clutch size σ^2^ = 0.000). a) Full model results and in bold, the model with the minimum AIC, and all models with a delta AIC<2 which were selected. b) Best fitting model(s) are shown with (averaged) parameter estimates and model weighting. Variables included in each model are marked with + and empty cells where variables were dropped. Results are shown using absolute clutch size and mean-centred clutch size.

**a)**

| **Intercept** | **Mean Individual Clutch Size** | **Population** | **Sex** | **df** | **logLik** | **AICc** | **delta** | **weight** |
| --- | --- | --- | --- | --- | --- | --- | --- | --- |
| ***Absolute clutch size*** | | | | | | | | |
| **-0.96** |  | **+** | **+** | **7** | **-587.33** | **1188.80** | **0.00** | **0.52** |
| **-0.97** | **0.02** | **+** | **+** | **8** | **-587.17** | **1190.60** | **1.74** | **0.22** |
| **-1.00** | **0.11** |  | **+** | **5** | **-590.29** | **1190.70** | **1.85** | **0.21** |
| -0.90 |  | + |  | 6 | -591.41 | 1194.90 | 6.12 | 0.03 |
| -0.94 | 0.11 |  |  | 4 | -594.08 | 1196.20 | 7.39 | 0.01 |
| -0.90 | 0.03 | + |  | 7 | -591.23 | 1196.60 | 7.80 | 0.01 |
| -1.00 |  |  | + | 4 | -600.76 | 1209.60 | 20.76 | 0.00 |
| -0.94 |  |  |  | 3 | -604.34 | 1214.70 | 25.88 | 0.00 |
| ***Mean-centred clutch size*** | | | | | | | | |
| **0.63** |  | **+** | **+** | **7** | **122.82** | **-231.50** | **0.00** | **0.69** |
| **0.63** | **0.00** | **+** | **+** | **8** | **122.85** | **-229.50** | **1.97** | **0.26** |
| 0.59 | 0.02 |  | + | 5 | 118.29 | -226.50 | 4.99 | 0.06 |
| 0.59 |  |  | + | 4 | 113.06 | -218.10 | 13.41 | 0.00 |
| 0.66 |  | + |  | 6 | 114.60 | -217.10 | 14.40 | 0.00 |
| 0.65 | 0.01 | + |  | 7 | 114.67 | -215.20 | 16.30 | 0.00 |
| 0.62 | 0.02 |  |  | 4 | 110.35 | -212.70 | 18.84 | 0.00 |
| 0.62 |  |  |  | 3 | 105.11 | -204.20 | 27.30 | 0.00 |

b)

| **Parameter** | **Parameter Estimate** | | **Standard Error** | | **Parameter Estimate** | **Standard Error** |
| --- | --- | --- | --- | --- | --- | --- |
|  | | **Absolute clutch size** | | | **Mean-centred clutch size** | |
| Intercept^1^ | | -0.97 | | 0.06 | 0.63 | 0.02 |
| D-Rouviere | | 0.08 | | 0.08 | -0.01 | 0.02 |
| E-Muro | | -0.14 | | 0.11 | -0.09 | 0.03 |
| E-Pirio | | -0.11 | | 0.09 | -0.06 | 0.02 |
| Mean Individual Clutch Size | | 0.03 | | 0.05 | 0.00 | 0.01 |
| Sex - Male | | 0.13 | | 0.05 | 0.06 | 0.01 |

^1^D-Muro is the population of reference and females the sex reference.

**Supplementary material: Appendix 7: Exploring Fourier modes in relation to clutch size**


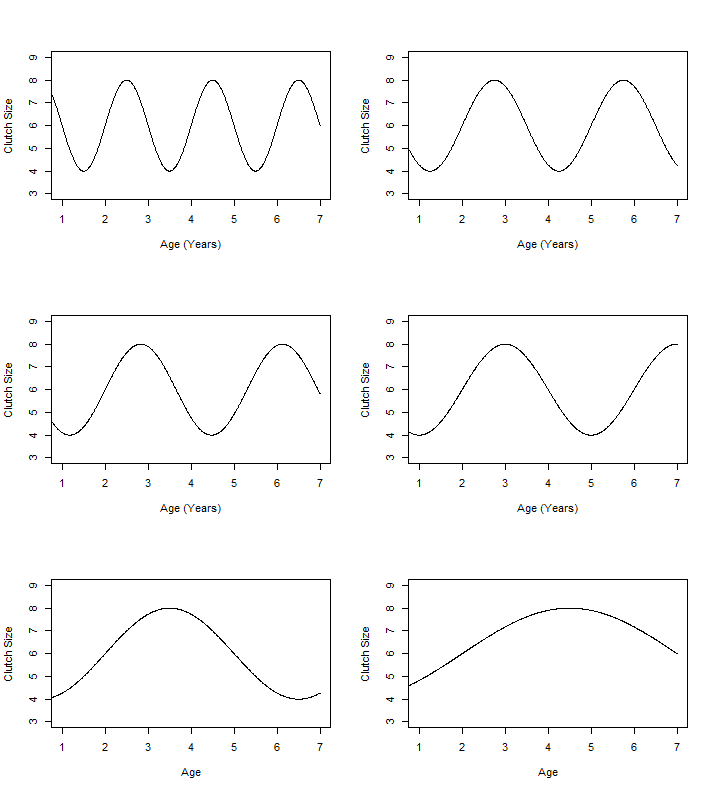


e)

f)

d)

c)

b)

a)

## Figure G1: Plots of each wavelength, extracted from the Fourier analysis, to visualise all reproductive investment curves (RICs) lengths detected in >1% of individuals in this study. An example individual is shown with a longevity of 7 years and clutch size range from 4 to 8 (amplitude = 2). a) Wavelength = 2; b) Wavelength = 3; c) Wavelength = 3.3; d) Wavelength = 4; e) Wavelength =6; f) Wavelength = 10.


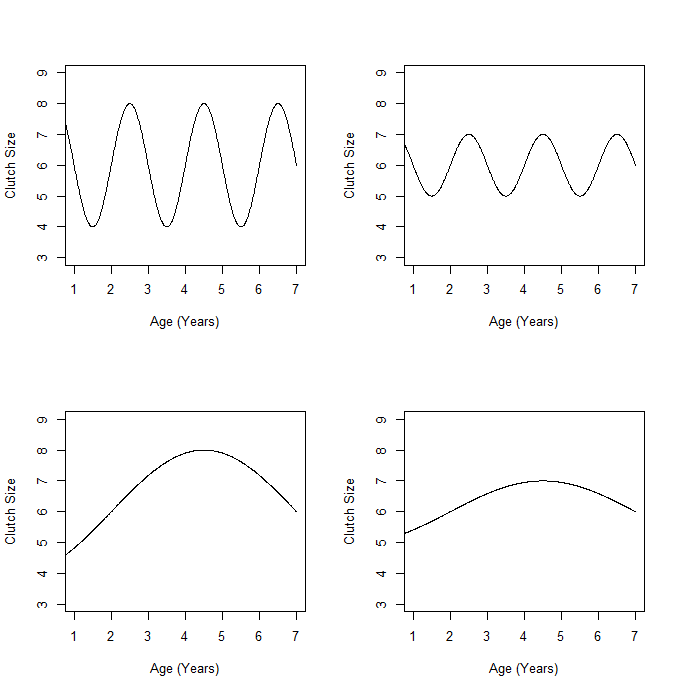


c)

d)

c)

d)

b)

a)

## Figure G2: Plots of wavelengths 2 and 10 to visualise how changes in the amplitude of the wavelength link to variation in clutch size. a) Wavelength = 2, amplitude = 2; b) Wavelength = 2, amplitude = 1; c) Wavelength = 10, amplitude = 2; d) Wavelength = 10, amplitude = 1.


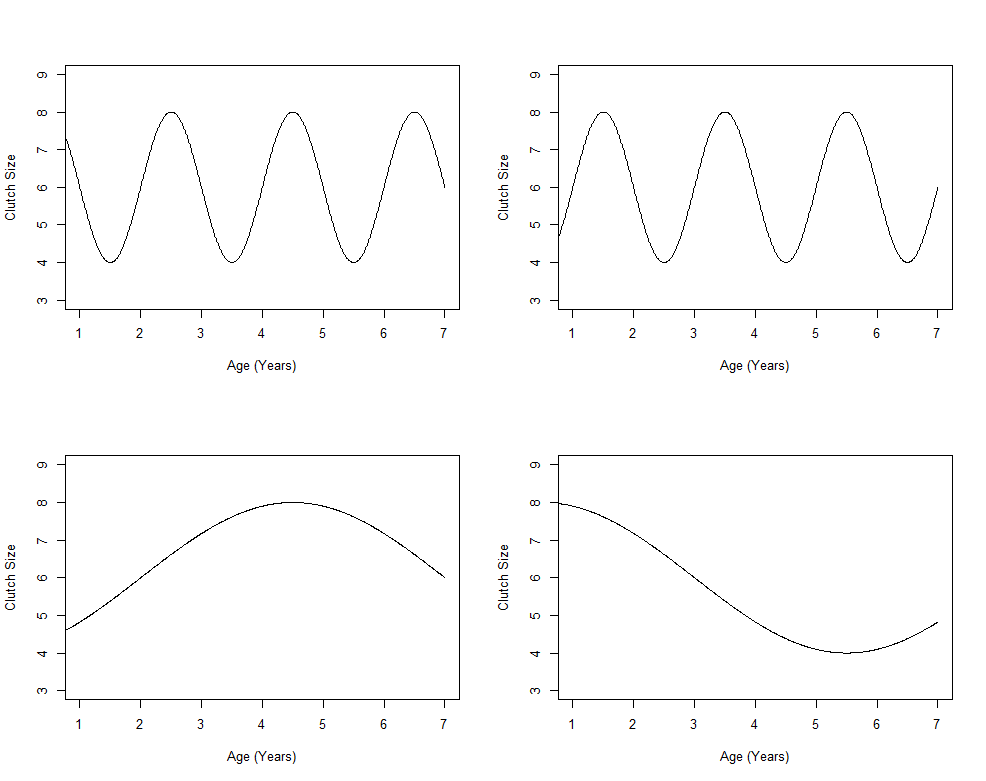


a)

b)

c)

d)

## Figure G3: Plots showing directionality in reproductive investment curves (RICs). Every Fourier mode shows a RIC but not necessarily the same direction – i.e. where the peaks and troughs of the wave are. Plots of wavelengths 2 and 10 to visualise how shifts in the peak of the wave change the age at which peak clutch size occurs. A) and b) Wavelength = 2 but maximum and minimum clutch sizes occur at opposite times c) Wavelength = 10 where peak reproduction occurs at 4/5 years showing a gradual increase over time then a decline in late life d) Wavelength = 10 but peak reproduction occurs at the first reproductive attempt with a steady decline over time. There is a slight increase in late life, possibly indicative of terminal investment. It is important to note the RICs occur over the same temporal scale in a and b, and c and d, but the peak reproduction occurs at different times.
